# A Recent Whole-Genome Duplication Divides Populations of a Globally Distributed Microsporidian

**DOI:** 10.1093/molbev/msw083

**Published:** 2016-04-27

**Authors:** Tom A. Williams, Sirintra Nakjang, Scott E. Campbell, Mark A. Freeman, Matthías Eydal, Karen Moore, Robert P. Hirt, T. Martin Embley, Bryony A. P. Williams

**Affiliations:** ^1^Institute for Cell and Molecular Biosciences, Newcastle University, Newcastle upon Tyne, United Kingdom; ^2^Biosciences, College of Life and Environmental Sciences, University of Exeter, Devon, United Kingdom; ^3^Ross University School of Veterinary Medicine, St. Kitts, West Indies; ^4^Institute for Experimental Pathology, University of Iceland, Keldur, Iceland

**Keywords:** Microsporidia, reductive genome evolution, gene birth, population genomics.

## Abstract

The Microsporidia are a major group of intracellular fungi and important parasites of animals including insects, fish, and immunocompromised humans. Microsporidian genomes have undergone extreme reductive evolution but there are major differences in genome size and structure within the group: some are prokaryote-like in size and organisation (<3 Mb of gene-dense sequence) while others have more typically eukaryotic genome architectures. To gain fine-scale, population-level insight into the evolutionary dynamics of these tiny eukaryotic genomes, we performed the broadest microsporidian population genomic study to date, sequencing geographically isolated strains of *Spraguea*, a marine microsporidian infecting goosefish worldwide. Our analysis revealed that population structure across the Atlantic Ocean is associated with a conserved difference in ploidy, with American and Canadian isolates sharing an ancestral whole genome duplication that was followed by widespread pseudogenisation and sorting-out of paralogue pairs. While past analyses have suggested *de novo* gene formation of microsporidian-specific genes, we found evidence for the origin of new genes from noncoding sequence since the divergence of these populations. Some of these genes experience selective constraint, suggesting the evolution of new functions and local host adaptation. Combining our data with published microsporidian genomes, we show that nucleotide composition across the phylum is shaped by a mutational bias favoring A and T nucleotides, which is opposed by an evolutionary force favoring an increase in genomic GC content. This study reveals ongoing dramatic reorganization of genome structure and the evolution of new gene functions in modern microsporidians despite extensive genomic streamlining in their common ancestor.

## Introduction

The Microsporidia are a major group of obligate endoparasitic fungi (Vavra and Lukes 2013) that cause economically important diseases of fish, edible crustacea (Kent et al. 1989; Campbell et al. 2013; Stentiford et al. 2013) and insects (Cornman et al. 2009; Pan et al. 2013), and serious opportunistic infections in immunocompromised humans (Hollister et al. 1996; Didier and Weiss 2011). In addition to a significant body of cell biological work aimed at understanding their unique adaptations to energy parasitism (Williams et al. 2002; Tsaousis et al. 2008), the Microsporidia have also become pre-eminent models for exploring the limits of reductive genome and cellular evolution in eukaryotes (Katinka et al. 2001). At 2.25 Mb, the microsporidian *Encephalitozoon intestinalis* has the smallest endoparasitic nuclear genome reported to date (Corradi et al. 2010). This reduction has been driven not only by host dependency and the loss of metabolic pathways associated with a free-living lifestyle, but also by a drastic compaction of classical eukaryotic genome architecture. The ∼1,800 protein-coding genes on the *E. intestinalis* genome are separated by highly reduced intergenic regions that have almost entirely lost promoters and regulatory elements; transcription of neighboring genes is often overlapping, suggesting that these motifs have moved within coding sequences in many cases (Williams et al. 2005).

With the increasing availability of genome sequences from diverse microsporidian lineages, it has now become clear that the highly compacted genomes of *E.* *intestinalis* and its relatives are not necessarily typical for the Microsporidia as a whole. Microsporidians with substantially larger genomes are phylogenetically dispersed across the group, with the largest genome to date (51.3 Mb) predicted for the mosquito pathogen *Edhazardia aedis* (Desjardins et al. 2015). Comparative analyses indicate that these differences in genome size are largely attributable to variation in the quantity of intergenic rather than protein-coding sequence (Heinz et al. 2012; Nakjang et al. 2013). As a result, some larger microsporidian genomes are actually less gene-dense than those of free-living eukaryotes such as *Saccharomyces cerevisiae* (Heinz et al. 2012), despite encoding far fewer genes. However, the evolutionary mechanisms underlying the variation in microsporidian genome size and intergenic content remain unclear; unlike in some other eukaryotic lineages, variation in the abundance of transposons and other selfish DNA elements does not appear to play a major role, as these make up relatively small proportions of even the largest microsporidian genomes (Heinz et al. 2012; Campbell et al. 2013). Comparative analyses have also revealed that microsporidian genome evolution has involved not only the loss of ancestral gene families, but also the gain of new, lineage-specific genes, some apparently by *de novo* origination from noncoding sequence (Carvunis et al. 2012; Nakjang et al. 2013). These genes comprise a significant proportion (19–52%; Nakjang et al. 2013) of the coding capacity of annotated microsporidian genomes, but their roles in parasite biology and lineage-specific adaptation remain unclear because they bear no recognizable similarity to characterized genes from model organisms.

To gain insight into these fundamental aspects of microsporidian biology, we initiated the broadest study to date of within- and between-population diversity for a globally distributed microsporidian, comprising isolates from the genus *Spraguea* that parasitise the goosefish (also known as monkfish; *Lophius* spp.). This genus includes the described species *Spraguea lophii*, infecting European goosefish, *Spraguea americana*, infecting American goosefish and *Spraguea gastrophysus* infecting the blackfin goosefish found in the West Atlantic. These three species are almost identical at the level of rDNA sequence and phylogenies using this gene do not recover clades corresponding to these different species (Casal et al. 2012; Yokoyama et al. 2013). For this reason, and for the purpose of this study, we consider *Spraguea* as a single evolutionary unit. *Spraguea* is an excellent model for microsporidian population genomics for several reasons. First, *Spraguea* infections result in the formation of spore-filled cysts (“xenomas”) which can be readily identified on the goosefish host and provide a plentiful supply of parasite DNA for sequencing. Second, *Spraguea* infections have been reported in goosefish (*Lophius piscatorius*) and other members of the genus *Lophius* throughout the world, enabling us to investigate the biogeography of a globally distributed parasite. Finally, the *Sp.* *lophii* reference genome, which we recently sequenced (Campbell et al. 2013), is relatively large by microsporidian standards (6 Mb) and contains longer intergenic regions, transposons, and a mixture of ancestral and lineage-specific genes, allowing us to investigate the evolutionary dynamics of each of these types of sequence for Microsporidia.

## Results and Discussion

### A Global Sampling of *Spraguea* Genomic Diversity

**Fig. 1:**
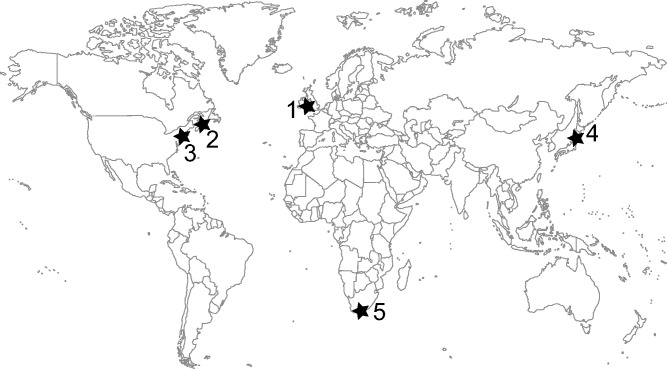
Geographical sampling of *Spraguea* isolates. Cysts were extracted from two to four fish at each of five locations worldwide. Site 1—Celtic Sea isolates (ex *L. piscatorius*): Celtic Deep (51.1921, Long. −5.6975), EM120 (West Lundy, 51.17535, −5.40448), RA12034 (Carmarthen Bay, 51.5459, −4.5872), North Atlantic (exact location unknown). Site 2—New Brunswick, Canada (ex *Lophius americanus*). Site 3 (ex *Lophius americanus*): JS61 (South of Pt. Judith, Rhode Island, USA, 40.9192, −71.7533), RM18 (Mud Hole, New Jersey, USA, 40.16666, −73.6666), RW92 (East of Ocean City, Maryland, USA, 37.9166, −74.7533). Site 4 (ex *Lophiomus setigerus*): Fukushima, Japan. Site 5 (ex *Lophius vomerinus*): Cape Town, South Africa. DNA was extracted from individual cysts, and each isolate was sequenced independently. A lack of evidence for heterozygosity on any of the genome assemblies suggests that each cyst arises from infection by a single haploid spore, or from a small clonal population.

We isolated and sequenced DNA from spores extracted from two to four cysts in each of five geographically distinct sampling locations: the Celtic Sea (from which we also obtained the material sequenced in the original *Sp.* *lophii* genome project; Campbell et al. 2013); the Bay of Fundy, New Brunswick, Canada; New Jersey, USA; Fukushima, Japan; and Cape Town, South Africa (fig. 1). Parasite genomes were sequenced to high coverage (mean 129*x* ± 62.5 SD, range 67–238*x*, see table 1) and independently assembled *de novo* (see Materials and Methods section). Because short-read sequencing technology has improved since the analysis of the *Sp.* *lophii* reference genome (Campbell et al. 2013), some of these new assemblies are of higher quality, both in terms of completeness and scaffold length, than the original reference (see table 1). We therefore used one of the new Celtic Sea isolates (“Celtic Deep”) as our reference genome for subsequent analyses. A recent study of the *Sp. lophii* karyotype indicates that the haploid genome contains 15 chromosomes ranging in size from 215 to 880 kbp (Mansour et al. 2013); based on these size estimates, some of the larger contigs in the Celtic Deep assembly likely represent entire chromosomes (largest contig 315 kbp, N50 103 kbp). Based on gene content comparisons to the published reference genome (supplementary table 1, Supplementary Material online), we obtained largely complete assemblies for 11 of our *Spraguea* isolates from the Celtic Sea and North American populations. We were unable to obtain high-quality *de novo* assemblies for either the South African or Japanese isolates, likely due to the lower quantities of parasite DNA extracted from these samples. In these cases, we still obtained sufficient parasite sequence data for mapping to the Celtic Deep reference (mean coverage 4.3*x* ± 2.4 SD, range 2.2–7.7*x*), for variant calling, and for allele frequency-based tests of population differentiation.

**Table 1:** Assembly Statistics for *De Novo* Population Genomes and the Published *Sp.* *lophii* Reference (Campbell et al. 2013).

Population	Isolate	Sequencing round	Assembly Size (bp)	N50 (bp)	Number of contigs	Largest contig (bp)	Mean coverage	Repeat content (%)
N/A	Campbell et al. (2013)	N/A	4,980,876	5,952	1,392	46,788	70x	4.02
Celtic Sea	Celtic Deep	1	5,774,772	102,889	295	314,819	127x	3.68
Celtic Sea	EM120	1	6,118,728	37,363	547	233,484	75x	3.73
Celtic Sea	RA12034	1	5,811,366	99,773	281	330,512	100x	3.64
Celtic Sea	North Atlantic	2	5,860,603	98,687	325	302,092	220x	3.62
North America	New Brunswick (NB) 1	1	7,734,566	8,814	2,491	73,309	82x	3.98
North America	NB4	1	7,741,808	9,066	2,459	73,272	91x	4.03
North America	NB8	1	7,758,278	8,988	2,456	73,323	67x	3.89
North America	NB9	1	7,712,656	9,052	2,399	80,777	104x	3.98
North America	New Jersey (NJ) JS61	2	7,753,355	5,453	2,859	70,878	112x	4.02
North America	NJ RM18	2	7,719,948	5,557	2,765	70,860	238x	3.98
North America	NJ RW92	2	7,706,796	5,588	2,722	71,120	213x	3.87

Note.—Population assignments are on the basis of our analysis of population structure and sampling location. *De novo* assemblies for North American isolates obtained from two different regions (New Brunswick and New Jersey) were much less contiguous than those for the Celtic Sea population, despite independent sampling, DNA extraction and sequencing procedures (sequencing rounds 1 and 2, see main text for discussion).

### A Whole-Genome Duplication in the Common Ancestor of North American *Spraguea* Isolates

One of the most striking differences between the North American and Celtic Sea assemblies was a difference in the number and length of assembled contigs; in comparison to the Celtic Sea isolates, which averaged 5.89 Mb (range 5.77–6.12 Mb), the North American assemblies all contained an additional ∼2 Mb of sequence divided across a large number of short contigs (table 1). These differences are unlikely to represent sequencing artifacts, because isolates from the two locations comprising the North American population were sampled by different workers and sequenced in separate sequencing rounds, each of which also contained members of the Celtic Sea clade (table 1).

Over large evolutionary distances, size differences in microsporidian genomes have sometimes been explained by changes in the amount of noncoding material, including the gain or loss of transposable elements (Kidwell 2002). That did not appear to be the case here because, while there are population-specific differences in transposon content (supplementary fig. 1, Supplementary Material online), the total amount of repetitive DNA in the Celtic Sea and North American genomes was similar overall, at ∼4% (table 1). Instead, our analyses revealed that variation in coding sequence was responsible for much of the observed size difference between the North American and Celtic Sea populations. Annotation of the North American genomes revealed a substantial number of duplicate gene pairs in which one of each pair contained a pseudogenizing frameshift mutation (range 258–400, median 370) relative to the Celtic Sea isolates (supplementary table 2, Supplementary Material online). We first evaluated the possibility that these frameshifts might represent assembly artifacts, although 80 of them are conserved across all independently sequenced and assembled North American isolates, and 344 are found on at least two of the North American assemblies. As an added control, we therefore tested, and confirmed, the presence of the frameshifts in four of these genes by PCR and Sanger sequencing (supplementary figs. 2 and 3, Supplementary Material online). Taken at face value, these observations suggested that many genes in the North American isolates have experienced a process of gene duplication followed by pseudogenization of one of the resulting copies (supplementary fig. 2, Supplementary Material online).

**Fig. 2:**
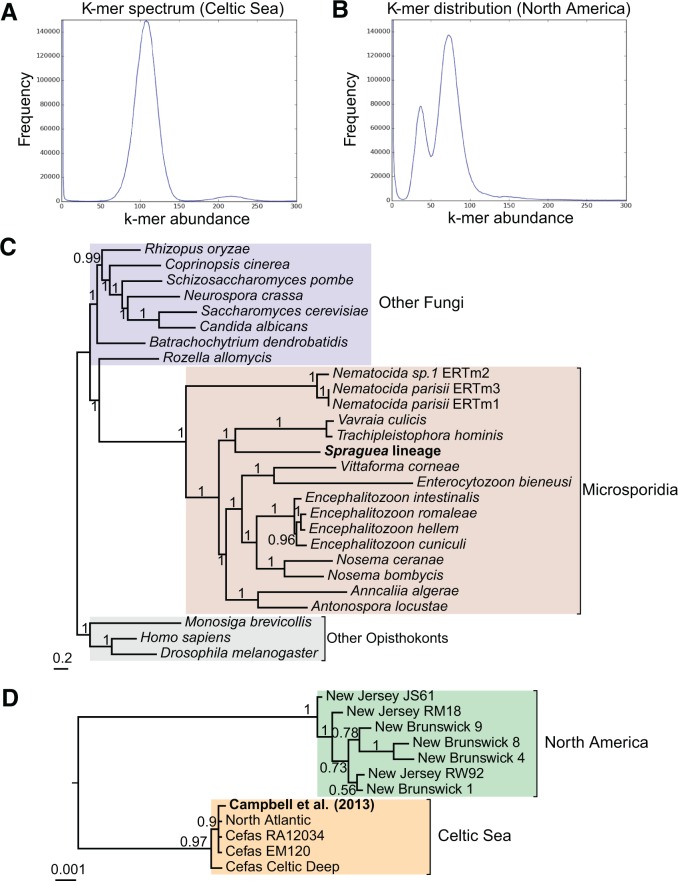
Evidence for a whole-genome duplication at the base of the North American clade. (*A*) The unimodal *k*-mer distribution for a representative Celtic Sea genome, that of the “Celtic Deep” isolate. (*B*) The *k*-mer distribution for a representative North American genome, the “New Brunswick 4” isolate. This distribution is bimodal, with peaks at 37× and 74×, suggesting the presence of both homozygous and heterozygous positions in North American genomes, but only homozygous positions in the Celtic Sea population. (*C*) A Bayesian phylogeny of the *Spraguea* lineage in the context of its microsporidian relatives and other opisthokont outgroups. Within-*Spraguea* relationships are collapsed due to the relatively short branch lengths in this region of the tree. (*D*) Relationships among *Spraguea* isolates, rooted using the tree depicted in (*C*). The earliest divergence splits the North American and Celtic Sea isolates with maximal posterior support (PP = 1), placing the ploidy increase at the base of the North American clade. These trees were inferred under the CAT + GTR model (Lartillot and Philippe 2004) in PhyloBayes-MPI 1.5a (Lartillot et al. 2013) using a concatenation of 23 single-copy orthologous protein-coding genes that share a congruent phylogenetic signal as determined by a hierarchical likelihood ratio test (Leigh et al. 2008).

We reasoned that these observations could be explained by either segmental or whole-genome duplication in the North American genomes, followed by pseudogenization of some of the duplicate gene copies. To distinguish between these possibilities, we compared the read depth (in terms of *k*-mer abundances) across both the North American and Celtic Sea assemblies (fig. 2), as well as the distribution of duplicate genes across the contigs of the North American assemblies. Duplicated, pseudogenized genes were distributed randomly across the *Spraguea* genome (supplementary fig. 4, Supplementary Material online), suggesting the action of a genome-wide process. The distributions of k-mer abundances are remarkably different between the two populations (fig. 2*A* and *B*): all of the Celtic Sea assemblies show a unimodal distribution, with abundance peaking at a coverage of roughly 100× (fig. 2*A*). In contrast, the North American assemblies all show a bimodal distribution, with peaks at *x* and 2*x* coverage; for example, the New Brunswick 4 k-mer distribution shows peaks at 37× and 74× coverage (fig. 2*B*). The simplest interpretation of these data is that there are two kinds of nucleotide site in the North American genomes: sites that are homozygous across the two copies of the genome (at 74× in the case of New Brunswick 4), and heterozygous sites (each at 37× coverage in New Brunswick 4), while there is only one kind of site in the Celtic Sea genomes. This observation strongly suggests that the genome copy number (i.e., the number of nonidentical genomes) of the North American isolates is twice that of the Celtic Sea population; haploidy and diploidy are most likely because we did not observe any evidence for three or more nucleotides at a given site in either population. The presence of a large number of additional short contigs in the North American genomes (supplementary fig. 5*A* and *B*, Supplementary Material online) is consistent with this hypothesis, because these may correspond to heterozygous regions poorly resolved by the assembly software.

To determine whether the increase of ploidy in the North American isolates resulted from a single event, we inferred a Bayesian phylogenetic tree based on 23 orthologous genes conserved as single copies across our isolates, their microsporidian relatives, and other opisthokont outgroups (fig. 2*C*). This tree shows a clear separation between the North American and Celtic Sea clades (fig. 2*D*), with all 11 isolates falling into one of two clearly distinct groups. Combined with the conservation of 80 of the observed pseudogenization events across all North American genomes, this result implies a single whole-genome duplication in the ancestor of the North American clade followed by an ongoing process of “sorting out” of the resulting paralogues, as has previously been reported for the palaeopolyploid yeast *S.* *cerevisiae* (Scannell et al. 2006, 2007).

### Population Structure, Ploidy and Spore Morphology

These findings also confirm that *Spraguea* populations are structured by geography, with a conserved difference in ploidy dividing populations across the Atlantic Ocean. To determine whether this geographic structure extends to the South African and Japanese isolates, for which we did not obtain enough material for a *de novo* genome assembly, we used the exact G-test (Raymond and Rousset 1995; Rousset 2008), a contingency table test that compares the distribution of allele (SNP) frequencies among the four sampled populations (North America, Celtic Sea, South Africa, and Japan). The result was highly significant (*P* = 0), indicating that the sampled *Spraguea* populations are structured by geography. This result stands in contrast to a recent population genomic analysis of another polyploid microsporidian, *Nosema ceranae* (Pelin et al. 2015), where eight populations from around the globe showed little evidence of geographic structure. *N. ceranae* infects honeybees, and the authors proposed that this lack of structure might reflect commercial exchange of infected bees for honey production. In contrast, our observation of population structure in *Spraguea* makes sense in light of the biology of the goosefish (*Lophius* spp.) hosts, which are bottom-dwelling fish that inhabit coastal waters at depths of up to 1,000 m (Hislop et al. 2001); intercontinental dispersal appears unlikely, although to our knowledge this question has not been addressed in detail. Recent work suggests that cannibalism of smaller goosefish by their larger conspecifics may represent an important mode of parasite transmission rather that ingestion of spores by an intermediate host or dispersal through the water column (Freeman et al. 2011). Combined with the endemism of the goosefish host, this mode of transmission provides a potential explanation for the strong geographic structure of *Spraguea* genetic diversity that we observe. Taken together with previous work, our analyses suggest that host biology is likely an important factor determining the extent of population structure in *Spraguea* and potentially other microsporidians.

Our findings are also interesting in that *Spraguea* spores isolated from fish in different parts of the world have been reported to show morphological differences. Spores from European and Tunisian isolates are dimorphic, with both uninucleate and dinucleate spores isolated from the same cyst, while spores isolated from American and Japanese fish are consistently uninucleate (Takvorian and Cali 1986; Freeman et al. 2004; Casal et al. 2012). Our microscopic observations of sampled spores showed that isolates from coastal North America were consistently uninucleate, while examined Celtic Sea isolates displayed a dinucleate state (supplementary fig. 6, Supplementary Material online). Recent work has provided karyotypic evidence that the double nuclei of spores from Tunisian *Spraguea* isolates contain identical copies of a haploid genome (Casal et al. 2012; Mansour et al. 2013). Taken together, these observations support a perhaps surprising scenario in which it is the single nuclei of North American spores which contain two divergent copies of the *Spraguea* genome, while the dinucleate spores of Celtic Sea isolates are genetically uniform.

### The Fate of Duplicate Genes Following Whole-Genome Duplication

**Fig. 3:**
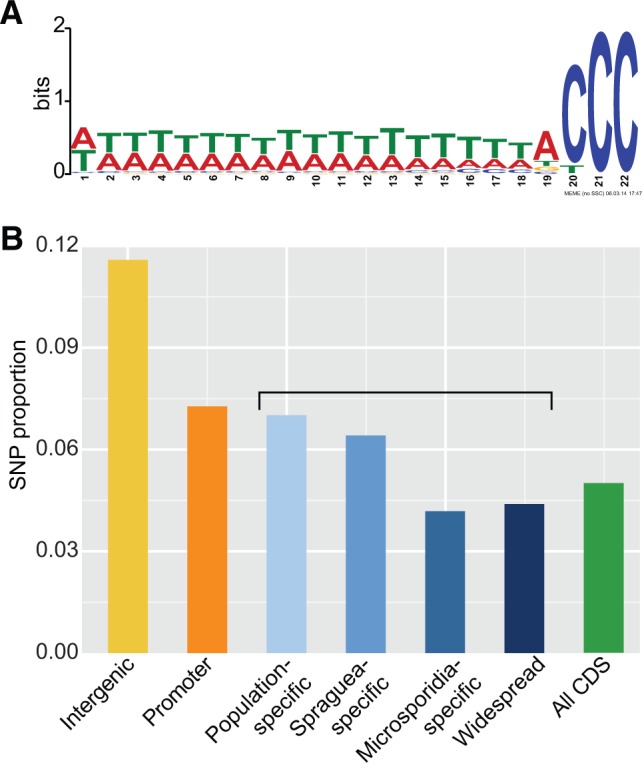
Selective constraint on *Spraguea* regulatory elements and protein-coding genes. (*A*) An enriched sequence motif found upstream of 1,439 out of 3,172 genes on the Celtic Deep reference genome. This motif is similar to those described immediately upstream of genes in a number of other Microsporidia, suggesting it may form part of a core microsporidian promoter. (*B*) Relative selective constraint (frequency of segregating SNPs) across intergenic regions, promoters, and protein-coding sequences on the Celtic Deep genome. Coding sequences are classified according to their taxonomic distribution, from those found only in one *Spraguea* population (“population-specific”) to those broadly conserved in eukaryotes.

The divergence between the *Spraguea* populations in North America and the Celtic Sea is relatively recent, with an average of 3% between-population silent-site divergence (see below). The changes that have occurred in the North American clade since that time therefore provide a fascinating window into the divergence between two genome copies at an early stage of the process. To investigate further, we evaluated the fate of duplicate gene pairs arising in the common ancestor of the North American population, defining three classes of genes: those for which both duplicates are retained in contemporary North American isolates (class A, 112 duplicate pairs, or 7% of the total), those in which one of the duplicates has been pseudogenized (class B, 980 pairs/68%), and those where both copies are now pseudogenes (class C, 358 pairs/25%). In these analyses, we defined open reading frames (ORF) with a length <85% of their Celtic Deep orthologs as pseudogenes; note that the total number of duplicate pairs (1,450) is less than the number of predicted protein-coding genes on the Celtic Deep reference genome because we restricted this analysis to cases where gene family relationships could be confidently assigned by MCL clustering (supplementary table 2, Supplementary Material online). As suggested by our initial PCR experiments, eventual fragmentation of one paralogue (class B, 68% of cases) was the most frequent outcome for genes arising from the whole-genome duplication, presumably restoring dosage and function to the pre-duplication state. The duplicates in class B (one pseudogenized duplicate) were not enriched for any functional category, consistent with a neutral process of duplication and subsequent loss. However, cases in which both gene pairs were pseudogenized following duplication were also reasonably frequent (25%). This observation is surprising in light of classical theory on the fate of duplicate genes, which typically posits loss of one of the duplicates, partitioning of ancestral functions between the duplicates by degeneration and complementation (i.e., subfunctionalization—Force et al. 1999), or the evolution of new functions for one member of the pair (neofunctionalization—Walsh 1995). Interestingly, this class C (both duplicates pseudogenized) was enriched for genes encoding microsporidia-specific and *Spraguea*-specific proteins, including leucine-rich repeat proteins and uncharacterized, lineage-specific genes (*P* = 0.002, hypergeometric test). Since *Spraguea*-specific genes, by definition, do not have detectable similarity to genes in other organisms, one possible explanation for the observed pattern of double-pseudogenizations is that these genes represent genome annotation artifacts that never had any function either pre- or post-whole genome duplication. Unfortunately, transcriptomic validation of gene models is challenging for *Spraguea* because we currently lack a cell culture system that would enable expression profiling across the parasite lifecycle. Nonetheless, our recent transcriptome analysis of a related microsporidian, *Trachipleistophora hominis* (Watson et al. 2015), demonstrated that the majority (73%) of species-specific genes were expressed at some stage of the parasite lifecycle, suggesting an important role for lineage-specific genes in microsporidian biology. Taken together with our findings that both species- and population-specific genes experience significant selective constraint above the background intergenic level (fig. 3), and that approximately half (49%) of class C genes do show recognizable similarity to genes in other organisms (supplementary table 2, Supplementary Material online), these results suggest that, while class C likely contains a higher proportion of artifactual gene models than classes A and B, at least some of the duplicate pairs that have undergone double pseudogenization were previously functional. One possibility is that these genes were members of functional categories that experience high rates of genomic turnover, such as those involved in host–parasite interactions. The inactivation of both copies of these genes following WGD might therefore reflect changes in effector protein repertoire resulting from host–parasite co-evolution during the evolution of the North American clade. An alternative possibility, discussed in more detail below, is that some of these genes may represent rapidly turned over “proto-genes” (Carvunis et al. 2012) which experienced different fates in the Celtic Sea and North American clades.

Cases in which both duplicates were retained (class A) comprise the smallest class (112 pairs, 7%), which was enriched for genes involved in transcription (KOG category K; *P* = 0.03, hypergeometric test), including transcription factors and RNA polymerases (supplementary table 2, Supplementary Material online). This result makes sense in light of previous work on dosage sensitivity (i.e., changes in relative expression levels) after gene duplication in yeast and other polyploids (Papp et al. 2003; Edger and Pires 2009), in which the copy numbers (and, therefore, expression levels) of core elements of the genetic system are maintained following WGD, perhaps to avoid the deleterious effects of dosage imbalances on the maintenance and regulation of core protein complexes in the cell. Overall, however, our finding that a substantial proportion of the observed duplication events—classes A and C, totaling 32% of duplicate families—resulted in a long-term change in gene content through either the retention, or the loss, of both duplicates suggests substantial functional divergence between the Celtic Sea and North American lineages.

### Selective Constraint on Regulatory Elements and Population-Specific Genes in *Spraguea*

A central issue in understanding the variation of genome size in Microsporidia, and indeed in other eukaryotes, is determining the proportion of each genome that is under selective constraint and likely to be functional. Scans using the MEME software (Bailey et al. 2009) revealed that the upstream regions of *Spraguea* genes contain a highly conserved promoter-like “CCC” motif (fig. 3*A*); found in 1,439 out of 3,172 upstream regions, (*E*-value for motif enrichment = 4.8 × 10^−^^1033^; median distance from translation start site: two bases) that is shared with other microsporidians (Peyretaillade et al. 2009). *Spraguea* genomes also encode substantial numbers of “hypothetical” protein sequences that may represent new genes that have recently arisen from noncoding sequence; such sequences are a common feature of microsporidian genomes, but their functions have been difficult to infer due to a lack of similarity to characterized genes from model organisms (Heinz et al. 2012; Nakjang et al. 2013). To compare selective constraint among intergenic regions, promoters, *de novo* genes and more conserved protein-coding sequences, we aligned the reads from each isolate to the Celtic Deep reference and compared the number of single nucleotide polymorphisms (SNPs) mapping to each category (fig. 3*B*); we then used Chi-squared tests to compare SNP frequency in each case, taking into account the total size of each category. The results provide new insights into the selective constraints operating on microsporidian genomes.

As expected, we find that the intergenic regions are experiencing the least selective constraint and observed SNPs in this category may approximate mutation rates. We then find that the putative promoter regions of *Spraguea* genes, which we define as the 22 bp upstream motif described above, are significantly (*χ*^2^ = 950.7, *P* = 9.17 × 10^−^^209^) conserved relative to the background intergenic level; this suggests that the regions immediately upstream of microsporidian coding sequences are enriched for functionally important regulatory elements, and is consistent with the idea that the “CCC” motif forms part of a core microsporidian promoter (Peyretaillade et al. 2009; Heinz et al. 2012). The heightened constraint acting just upstream of protein-coding genes in contemporary *Spraguea* populations parallels, and helps to explain, the retention of the “CCC” motif in microsporidia that have otherwise experienced a dramatic reduction in the length of their intergenic regions. This result also suggests that, although the intergenic regions of larger microsporidian genomes contain distal regulatory motifs (Heinz et al. 2012), these make a relatively small contribution to intergenic length. Much of the remaining sequence can be lost during reductive evolution without any obvious fitness costs to the organism, and in that sense might be regarded as nonfunctional or “junk” DNA.

Unsurprisingly, our analysis also confirmed that protein-coding sequences are significantly conserved above the background intergenic level (*χ*^2^ = 71332.19, df = 1, *P* = 0). However, it is interesting to note that this relationship held not only for broadly-conserved genes (i.e., genes which also had homologues outside the *Spraguea* lineage), but also for *Spraguea-*specific (*χ*^2^ = 14936.04, df = 1, *P* = 0) and population-specific (*χ*^2^ = 4293.46, df = 1, *P* = 0) genes, suggesting that both categories of genes are playing important roles in *Spraguea* biology. In particular, the conservation of population-specific genes (i.e., genes found in either the North American or Celtic Sea populations, but not in both) indicates that even recently derived *de novo* genes can make significant contributions to organismal fitness, and is suggestive of local adaptation to the specific goosefish host. Finally, we detected fewer SNPs in the *Spraguea* homologues of conserved microsporidia-specific genes than either population- or *Spraguea*-specific genes (*χ*^2^ = 2410.02, 2489.42, respectively; df = 1 and *P* = 0 for both comparisons), perhaps reflecting their fundamental roles in the parasitic lifecycle. Consistent with the increasing selective constraint we observe moving from lineage-specific to broadly conserved gene families, we also observed a higher proportion of nonsynonymous-to-synonymous SNPs segregating in the population-specific (0.19) and *Spraguea*-specific (0.17) genes compared with more widely conserved gene families (microsporidia-specific: 0.12, broadly conserved: 0.1). Note that these ratios are not directly comparable to standard dN/dS estimates of selective constraint, because negative selection may not yet have had time to filter out deleterious variants segregating within a population (Kryazhimskiy and Plotkin 2008).

### Mechanisms for the Origin of New Genes from Noncoding Sequence

**Fig. 4:**
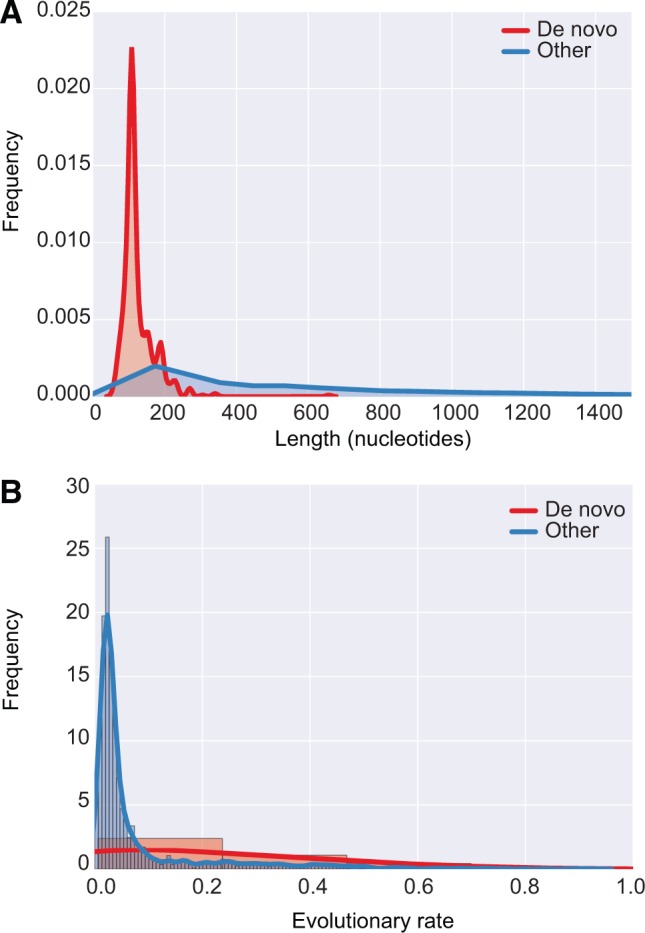
Recently-evolved, *de novo* genes are shorter than older genes in *Spraguea*, and experience more variable selective pressures. (*A*) Genes that have emerged since the divergence of the Celtic Sea and North American populations are significantly shorter than older genes (*P* = 1.2 × 10^−298^, Wilcoxon rank-sum test), consistent with their recent emergence from noncoding sequence. (*B*) Distribution of within-gene family evolutionary rates [calculated as mean pairwise Kimura 2-parameter genetic distances (Kimura 1980)] for *de novo* versus older gene families. Although the means of these distributions are similar (0.19 for *de novo*, 0.09 for older, *P* = 0.13, Wilcoxon rank-sum test), the variance of the rates for *de novo* genes is significantly greater (0.12 for *de novo* genes, 0.02 for older, *P* = 0.0005, Levene’s test). These results suggest that, while some *de novo* genes experience significant selective constraint (fig. 3), others are likely non-functional and subject to rapid turnover through evolutionary time.

The identification of a class of open reading frames found in one population but not the other was intriguing, and we investigated the evolutionary properties of these sequences to evaluate whether they represented new, *de novo* genes that had evolved since the divergence of the Celtic Sea and North American populations. To evaluate mechanisms for the origin of *de novo* genes, we investigated the properties of genes that were polymorphic in one of the two *Spraguea* populations, absent in the other, and which had no significant BLASTP hits to sequences in the NCBI nr database; that is, lineage-specific genes which have arisen since the divergence of the two populations. Our *Spraguea* genomes encode a total of 325 such families, each containing one or more population-specific genes. Interestingly, the coding sequences of roughly half of these families (172, 52%) show significant sequence similarity to noncoding regions from other *Spraguea* genomes. For these 172 *de novo* gene families, the mean percentage identity to noncoding regions is remarkably high (97.1% ± 3.4% identity, with mean coverage of 98% ± 0.04%), suggesting that *de novo* genes first arise by a small number of mutations that create an open reading frame from previously noncoding sequence, perhaps by addition of a start codon or elimination of a stop codon (see supplementary fig. 7, Supplementary Material online for illustrations of two examples). Consistent with an origin from noncoding sequence, the average length of new genes is significantly shorter than that of older, more widely conserved genes (mean length for *de novo* genes: 125 nucleotides; mean for other genes: 621 nucleotides; *P* = 2.43 × 10^−^^220^, Wilcoxon rank-sum test; see fig. 4*A*). Very few of these genes are expressed: of the 81 *de novo* genes segregating in the Celtic Sea population, only 13 had any detectable expression in the published *Spraguea* transcriptome (Campbell et al. 2013). Further, a comparison of evolutionary rates (fig. 4*B*) indicates that the evolutionary rates of *de novo* genes are both higher (Kimura 2-parameter evolutionary rate for *de novo* genes: 0.32; rate for other genes: 0.08; *P* = 0.007, Wilcoxon rank-sum test) and significantly more variable than those of older, more broadly-conserved genes (*P* = 1.38 × 10^−^^13^, Levene’s test, see fig. 4*B*), suggesting that these genes as a class experience lesser and more variable selective constraints than older genes. Thus, many of these genes likely represent nonfunctional ORFs that simply occur by chance in nongenic sequence. Nonetheless, the observation that this class as a whole is significantly conserved above the background intergenic level (fig. 3) suggests that at least some *de novo* ORFs may have already acquired selectively advantageous functions. One possibility is that these sequences are proto-genes (Carvunis et al. 2012), consistent with a model in which new genes begin as short, fortuitously expressed regions of previously noncoding sequence that can be retained and elaborated by selection if they initially provide a useful function.

### Evolution of Nucleotide Composition in Microsporidian Genomes

**Fig. 5:**
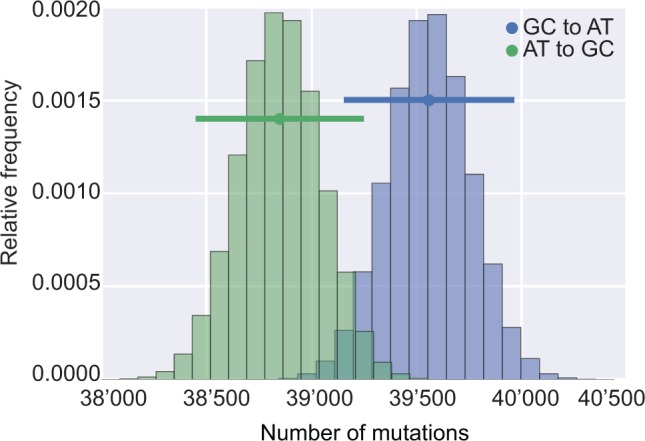
The AT content of the *Spraguea* genome is near mutational equilibrium. To investigate the mutation process in *Spraguea*, we focused on mutations segregating in the intergenic genomic regions. Although these regions are highly AT-rich (79.2%), we counted similar numbers of AT to GC and GC to AT mutations segregating in contemporary *Spraguea* populations, suggesting that these regions are near mutational equilibrium. The observed number of mutations in each direction is indicated by a dot; the bars represent 95% confidence intervals based on resampling from a Poisson distribution with mean equal to the observed number of mutations. Based on the method of Hershberg and Petrov (2010), we estimate an equilibrium AT content of 79.5% for these regions. Thus, the AT content of both the intergenic and, by extension, the coding regions of the *Spraguea* genome (which have a mean AT content of 74.7%) are close to mutational equilibrium.

Microsporidian genomes often show extremely skewed nucleotide compositions, and at 76.7% AT, the *Spraguea* genome is no exception. To determine whether this composition reflects an underlying mutational bias towards AT, we examined the SNPs segregating in our *Spraguea* populations. We counted the numbers of mutations in each direction segregating at intergenic sites in both the North American and Celtic Sea populations, using the method of Hershberg and Petrov (2010). We restricted our analysis to SNPs segregating in intergenic regions because these are experiencing the least measurable selective constraint (fig. 3), and therefore most closely reflect the mutational process. We found more GC to AT mutations segregating in both populations, although bootstrapped 95% confidence intervals for the observed counts overlap (fig. 5), implying this difference is not significant. This result suggests a mutational bias toward AT because, as the AT-content of the genome is already high, we would expect a higher number of mutations from A or T to G or C nucleotides given the higher numbers of A or T available for mutation; it also follows that an equal number of GC to AT and AT to GC mutations is indicative of a nucleotide content at mutational equilibrium. By taking into account the number of A/T and G/C sites on the *Spraguea* genome and the inferred directionality of the segregating mutations, we calculated the expected AT content at mutational equilibrium; this was 79.5%, very close to the current value of 79.2% for intergenic regions, and confirming that the composition of the intergenic sequences in *Spraguea* is close to mutational equilibrium. These observations are consistent with reported mutational biases to AT in a number of very different organisms, from bacteria (Hershberg and Petrov 2010) to model eukaryotes (Lynch et al. 2008; Lynch 2010); our evidence for the same bias in an intracellular eukaryotic parasite such as *Spraguea* supports the proposal that mutation is universally biased towards AT (Hershberg and Petrov 2010). If so, then the observed variation in microsporidian AT content (mean AT: 64.4%, range 52.6–76.7%) might result from the interaction of this mutational pressure with other evolutionary forces, such as natural selection.

**Fig. 6:**
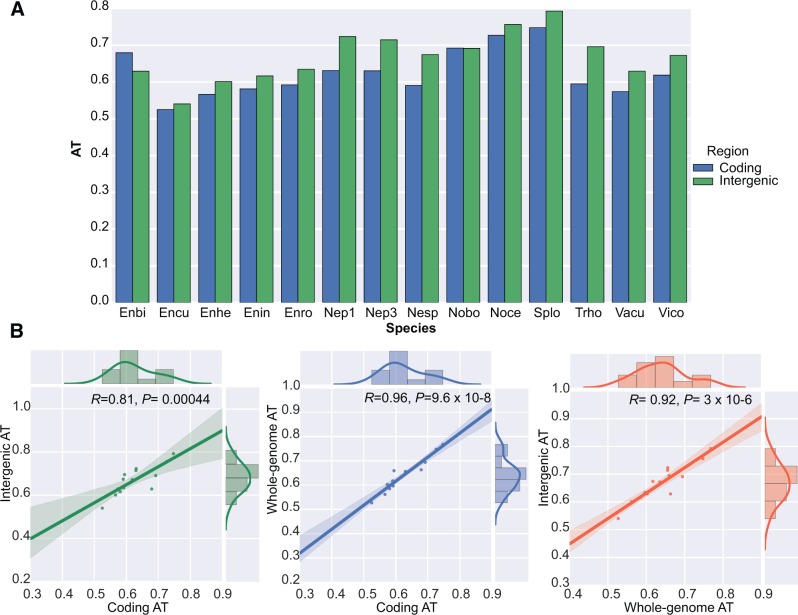
An evolutionary force favors lower AT content in microsporidian genomes. (*A*) In all microsporidian genomes analyzed except that of *E. bieneusi*, the AT content of intergenic regions exceeds that of coding sequences. This trend is significant across the whole dataset (*P* = 0.001199, paired *t*-test), as well as within each individual genome (*P* < 10–24 for all comparisons; see additional file 3, Supplementary Material online for details). These data suggest selection for more moderate nucleotide compositions in coding sequences, perhaps as a result of functional constraints. (*B*) Correlations between coding, intergenic and whole-genome AT content across the complete set of published microsporidian genomes. All three variables are highly and significantly correlated; in particular, the correlation between the AT content of coding and intergenic regions within each genome suggests that the selective pressure against AT extends to intergenic regions. This pattern might be explained by variation in the extent of biased gene conversion, which in eukaryotes tends to increase GC content, across the Microsporidia. The correlations reported here are robust to the underlying microsporidian phylogeny, as assessed by phylogenetic generalized least squares regression [*P* = 0.00049, *P* = 5.3 × 10^−7^, *P* = 6.9 × 10^−5^ for the three comparisons in (*b*), respectively].

As an initial test of the idea that selection favors lower AT content in microsporidian genomes, we compared the AT content of intergenic and coding sequences in genomes sampled from across the microsporidian radiation. AT content was significantly lower in coding than in intergenic regions for all the genomes we analyzed, with the exception of *Nosema bombycis* and *Enterocytozoon bieneusi* (fig. 6*A*); in the case of *E.* *bieneusi*, this may be due to the apparent inclusion of contaminant bacterial sequences with significantly different codon usage in the genome assembly (Heinz et al. 2012). Given that coding sequences experience significantly greater selective constraint than intergenic regions (fig. 3), these results suggest that the selective pressure against AT reported previously for Bacteria (Hildebrand et al. 2010) is also at work in the Microsporidia. Next, we evaluated the relationships between the AT content of microsporidian coding and intergenic regions on a per-genome basis (fig. 6*B*). Intriguingly, we found that coding and intergenic AT content was highly and significantly correlated (*P* = 0.00044): Microsporidia with lower AT content in their coding regions also tend to have intergenic regions than are less AT-rich. The correlation remains highly significant when accounting for phylogenetic structure (*P* = 0.00049), suggesting that it is a general feature of microsporidian genomes and not an artifact of biased taxonomic sampling. Thus, the evolutionary force acting against AT appears to operate genome-wide in Microsporidia, although with greater efficacy in coding regions.

There are at least three plausible candidates for the nature of this evolutionary force, which are difficult to distinguish based on current data: most simply, the strength of the mutational bias towards AT might vary across the Microsporidia, leading to differences in AT content at mutational equilibrium. Alternatively, variation in the strength of biased gene conversion, a form of heterologous recombination which in eukaryotes tends to promote the fixation of GC-rich sequences (Birdsell 2002), might vary across the group—particularly given that ploidy and reproductive mode are apparently quite labile during microsporidian evolution (this study and Pombert et al. 2013).

### Generalism, Specialism, and the Strength of Selection

A tempting, though elaborate, third hypothesis for explaining the nucleotide composition of microsporidian genomes is that lineage-specific differences in parasitic lifestyle, particularly differences in host range, might drive changes in the genome-wide intensity of selection. Microsporidians such as *Encephalitozoon* *cuniculi* are “generalists”, able to infect a broad range of host species, while *Spraguea* is apparently restricted to a single host (Didier et al. 2000). A generalist might be expected to have a larger population size than a highly host-restricted species, because of the greater number of potential hosts they can infect; this would tend to increase the efficacy of selection relative to mutation and drift in generalist lineages. To test this hypothesis, we compared levels of silent-site diversity in *Spraguea* both within and between populations to values reported in recent epidemiological studies of *E. cuniculi* (Peuvel et al. 2000; Xiao et al. 2001; Pombert et al. 2013). Interestingly, silent-site diversity among *E. cuniculi* isolates infecting rabbits, mice and dogs (0.0056/site—Pombert et al. 2013) was on the order of diversity *within* each *Spraguea* population (Celtic Sea: 0.001, North America: 0.008), each of which infects a related species in the same goosefish genus; divergence between the two *Spraguea* populations is much higher (0.03 SNPs/silent site). These values suggest that the *E. cuniculi* isolates infecting several different mammalian hosts (human, mouse, blue fox, rabbit, dog) are more homogeneous than the *Spraguea* isolates infecting two closely related fish species, providing some support for a generalist/specialist distinction between these two lineages, with potential implications for population size and the strength of selection. Population genomic sampling from other microsporidians that vary in these traits would permit a definitive test of this hypothesis, but such data are not yet available.

## Conclusions

Our population genomic analysis of *Sp.* *lophii* is the first such study for a microsporidian in its natural, wild host, and has provided valuable new insights into the genome biology and evolution of these diverse and highly successful intracellular parasites. We show that within this genus, highly similar SSU rRNA sequences belie large-scale genomic changes over a short evolutionary timescale. This situation is reminiscent of closely related plant species that have undergone recent polyploidization events with subsequent divergent genome rearrangements (Liu et al. 2014). Our analyses also revealed that genetic diversity in *Spraguea* is strongly structured by geography, demonstrating that, despite the very large numbers of spores that can arise from a single infection, parasite demography is tightly coupled to the population structure of the largely endemic goosefish host. Since obligate intracellular parasitism is a conserved feature of all microsporidians, our results suggest that population sizes may be severely limited by host demography in the group as a whole. This would imply relatively low population sizes for microsporidians compared with their free-living fungal relatives, but also important lineage-specific differences in population size between microsporidians infecting, for example, vertebrates (with relatively low population sizes) and insects (with larger population sizes). The effect of these differences on the strength of selection remains to be evaluated, but may provide new insight into the diversity of genome sizes and architectures observed for sequenced microsporidians.

Our most important result is the discovery of a relatively recent whole-genome duplication in the common ancestor of the North American *Spraguea* clade. While variation in levels of ploidy and heterozygosity have previously been reported over short evolutionary timescales in microsporidia (Haag et al. 2013), the degree of divergence, and the sorting-out of duplicate pairs, that has occurred in the North American *Spraguea* population is unprecedented for microsporidia, raising intriguing parallels with the process of whole-genome duplication in model eukaryotes such as yeast (Scannell et al. 2006, 2007) and vertebrates (McLysaght et al. 2002; Dehal and Boore 2005). This is particularly striking given that microsporidian genomes are typically highly reduced, with many otherwise broadly-conserved genes and cellular features lost in the common ancestor of the group (Williams et al. 2002; Tsaousis et al. 2008). Combined with our evidence for the evolution of new genes *de novo* from previously noncoding sequence, these findings imply that the same processes that generate evolutionary novelty in model eukaryotes—duplication, divergence, and *de novo* innovation—are at work in microsporidians, the most highly reduced parasitic eukaryotes described to date.

## Materials and Methods

### DNA Extraction and Sequencing

Celtic sea samples were collected during the CEFAS (*Centre for Environment, Fisheries and Aquaculture Science*, UK*)* 2012 UK marine sampling cruise and stored frozen until processing. They were then defrosted and purified by passing through a 70-µm cell strainer and further purified by passing through a percoll gradient as described in Campbell et al. (2013) and DNA was extracted by a phenol chloroform protocol as described in Campbell et al. (2013). New Brunswick and New Jersey samples were stored frozen in PBS and processed as above. Japanese samples were stored in ethanol and processed as above or using a Qiagen DNA mini kit (Qiagen, Venlo, the Netherlands). DNA libraries were sequenced on an Illumina HiSeq 2500, producing 2 × 250-bp paired-end reads for the first round of samples (Celtic Sea and New Brunswick isolates), and 2 × 300-bp paired-end reads for the second round of samples (Celtic Sea, New Jersey, South African, and Japanese isolates).

### *De* *Novo* Genome Assembly and Annotation

Adapter sequences and low-quality bases (*Q* <30) were detected and removed using Fastq-Mcf (Aronesty 2013). Trimmed, quality-filtered reads were error-corrected using Quake (Kelley et al. 2010). Based on the estimated 6.2–7.3 Mb size of the reference *Spraguea* genome (Campbell et al. 2013), the k-mer size for error correction was set to 15 (*k* = log(200 × size(bp))/log(4)∼ = 15.1); we also experimented with *k* = 16, and obtained very similar results. Error-corrected reads were assembled separately for each isolate using SPAdes 3.0 (Bankevich et al. 2012), with parameters recommended by the authors for the assembly of paired-end Illumina reads (spades.py −*k* 21,33,55,77,99,127—careful—only-assembler). As the best of these *de novo* assemblies (that belonging to the Cefas “Celtic Deep” isolate) was longer and more contiguous (N50: 102,899 bp; Largest contig: 314,819 bp; number of contigs: 295; total assembly size: 5,742,641 bp) than the original *Sp. lophii* genome assembly (Campbell et al. 2013), we used it as our reference genome for subsequent analyses. Distributions of k-mer abundances were calculated using khmer 1.4 (Crusoe et al. 2015); the results shown in figure 2*A* and *B* are for *k* = 23, although results were very similar for a range of plausible values (*k* = 15–25). We used Prodigal 2.60 (Hyatt et al. 2010) to predict open reading frames on each of our *de novo* assemblies, and obtained functional annotations for these by identifying their orthologs on the published, annotated *Sp.* *lophii* genome (Campbell et al. 2013). Although Prodigal was designed for gene finding on prokaryotic genomes, we have found that it outperforms standard eukaryotic gene finding algorithms for Microsporidia, perhaps because of the extreme paucity of introns and other typical features of eukaryotic gene architecture in microsporidian genomes, and it has also been successfully used in gene calling for other microsporidians (Cuomo et al. 2012). Comparisons of our *de novo* assemblies to the published reference genome, in terms of gene content and completeness, are provided in supplementary table 1, Supplementary Material online. New assemblies have been deposited at NCBI under Bioproject number PRJNA269798.

### Remapping and Variant Calling

In addition to inferring *de novo* assemblies for each isolate, we also mapped the filtered and error-corrected reads from each sample onto the “Celtic Deep” reference using Stampy (Lunter and Goodson 2011); overlapping paired-end reads were merged before alignment using FLASH (Magoc and Salzberg 2011). The resulting short-read alignments were de-duplicated using the MarkDuplicates module of the Picard command-line tools (http://broadinstitute.github.io/picard/, last accessed October 2015). SNPs and indels were called using samtools mpileup, filtering out variant calls with read depth <10 and quality scores <60.

### Phylogenetics and Analysis of Population Structure

Phylogenetic analyses were performed using a set of 23 microsporidian marker genes we have used previously (Nakjang et al. 2013), updated to include orthologs from the new *Spraguea* genomes using the Cognitor method (Tatusov et al. 2003). Sequences were aligned using Muscle (Edgar 2004), poorly aligning regions were removed using BMGE (Criscuolo and Gribaldo 2010), and phylogenetic analyses were performed under the CAT + GTR model in PhyloBayes-MPI 1.5a (Lartillot et al. 2013). Two chains were run, and convergence was assessed periodically using the included bpcomp and tracecomp programs. The chains were stopped, and a consensus tree inferred, when the maximum difference in bipartition frequencies (bpcomp) and a variety of continuous parameters (tracecomp) between the chains was <0.1, with effective sample sizes >100 for all continuous parameters, as recommended by the authors.

Allele frequency-based tests of population differentiation were carried out using Genepop (Raymond and Rousset 1995), with populations defined *a priori* based on geographic origin (North America, Celtic Sea, South Africa, Japan). Isolates were genotyped at each variable site based on alignments of the sequencing reads from each isolate to the “Celtic Deep” reference, using the variant calling approach described above.

### Tranposon Similarity Networks

We used *E*-values derived from an all-versus-all BLASTP search of non-LTR retrotransposon sequences to build a similarity network using the BLAST2SimilarityGraph module in Cytoscape 2.8.2 (Saito et al. 2012); the visualization in supplementary figure 1, Supplementary Material online employs a force-directed layout. The three sequence clusters discussed in the text were insensitive to a range of increasingly stringent *E*-value cutoffs (10^−^^5^–10^−^^20^).

### Identification of Regulatory Motifs

Based on previous analyses of the *T. hominis* genome (Heinz et al. 2012), we extracted the first 50 bp upstream of each annotated protein-coding gene on the Celtic Deep genome, taking into account the coding strand and orientation of each gene; this resulted in a set of 3,172 upstream regions. We searched these regions for enriched motifs using the standalone version of MEME 4.9.1 (Bailey et al. 2009).

### Analysis of Selective Constraint

Our analysis of selective constraint was based on the remapping of sequencing reads from each isolate to the Celtic Deep reference genome. We used this remapping approach in order to make full use of the sequencing data from the South African and Japanese populations; note that one limitation of this approach is that only genes specific to the Celtic Sea population could be included in the population-specific test described below. That genome was divided into the following regions: intergenic (all intergenic regions, excluding the 50 bp upstream of each annotated protein-coding gene); promoter (the 22-bp upstream motif detected using the MEME search); and coding sequences. Coding sequences were further classified according to the taxonomic distribution of homologues of the gene product, based on BLASTP searches of the protein sequence against the NCBI nr database, at an *E*-value cutoff of 10^−^^5^. These categories were as follows: population-specific—genes with significant hits only in one *Spraguea* population; *Spraguea*-specific—genes found in all *Spraguea* populations, but in no other organisms; Microsporidia-specific—genes with significant hits in one or more other microsporidian genomes, but not outside the group; Widespread—genes with significant hits in one or more other microsporidians, plus one or more other eukaryotes. To estimate the relative levels of selective constraint, we calculated the total number of SNPs that map to each of these categories, and divided by the number of bases in that category. Since many SNPs were shared within individual populations, we counted multiple hits at the same site only once, to avoid overestimating the number of mutations. We used Chi-squared tests to compare the frequencies of SNPs in the different categories, implemented using the “chisq.test” function in R (http://www.r-project.org, last accessed January 2016).

### Statistical Analysis of Nucleotide Composition

The expected nucleotide composition at mutational equilibrium was calculated using the method of (Hershberg and Petrov 2010). We polarized the direction of observed mutations (GC to AT and vice-versa) under the assumption that the most frequent nucleotide represented the ancestral state. We used this frequency-based approach for polarizing mutations because it is unaffected by the high degree of inter-relative to intra-population divergence between the North American and Celtic Sea populations; the (relatively) long branch leading to the outgroup is expected to introduce bias into parsimony-based inferences of the ancestral state (Hildebrand et al. 2010). Bootstrapped 95% confidence intervals around the observed numbers of segregating GC to AT and AT to GC mutations were simulated using the random.poisson function in numpy (Oliphant 2007). Coding, intergenic and genome-wide AT content was calculated directly from the assemblies for published microsporidian genomes downloaded from MicrosporidiaDB (Aurrecoechea et al. 2011); the list of genomes included in our analysis is provided in supplementary table 3, Supplementary Material online. Linear regressions were performed and visualized using the Python packages matplotlib 1.3.1 (Hunter 2007) and seaborn 0.3.1 (http://www.stanford.edu/∼mwaskom/software/seaborn/, last accessed January 2016). Phylogenetic generalized least squares regressions were carried out using the R package “ape” (Paradis et al. 2004).

### Silent-Site Diversity

Silent-site diversity values were calculated from pairwise comparisons of the aligned intergenic regions for all pairs of within- and between-population *Spraguea* isolates from the North American and Celtic Sea populations.

## Supplementary Material

Supplementary figures 1–7 and tables 1–3 are available at *Molecular Biology and Evolution* online (http://www.mbe.oxfordjournals.org/).

Supplementary Data

## References

[msw083-B1] Aronesty E. 2013 Comparison of Sequencing Utility Programs. The Open Bioinformatics Journal. 7:1–8.

[msw083-B2] Aurrecoechea C Barreto A Brestelli J Brunk BP Caler EV Fischer S Gajria B Gao X Gingle A Grant G, 2011 AmoebaDB and MicrosporidiaDB: functional genomic resources for Amoebozoa and Microsporidia species. Nucleic Acids Res. 39:D612–D619. 20974635 10.1093/nar/gkq1006 PMC3013638

[msw083-B3] Bailey TL Boden M Buske FA Frith M Grant CE Clementi L Ren J Li WW Noble WS. 2009 MEME SUITE: tools for motif discovery and searching. Nucleic Acids Res. 37:W202–W208. 19458158 10.1093/nar/gkp335 PMC2703892

[msw083-B4] Bankevich A Nurk S Antipov D Gurevich AA Dvorkin M Kulikov AS Lesin VM Nikolenko SI Pham S Prjibelski AD, 2012 SPAdes: a new genome assembly algorithm and its applications to single-cell sequencing. J Comput Biol. 19:455–477. 22506599 10.1089/cmb.2012.0021 PMC3342519

[msw083-B5] Birdsell JA. 2002 Integrating genomics, bioinformatics, and classical genetics to study the effects of recombination on genome evolution. Mol Biol Evol. 19:1181–1197. 12082137 10.1093/oxfordjournals.molbev.a004176

[msw083-B6] Campbell SE Williams TA Yousuf A Soanes DM Paszkiewicz KH Williams BA. 2013 The genome of *Spraguea lophii* and the basis of host-microsporidian interactions. PLoS Genet. 9:e1003676. 23990793 10.1371/journal.pgen.1003676 PMC3749934

[msw083-B7] Carvunis AR Rolland T Wapinski I Calderwood MA Yildirim MA Simonis N Charloteaux B Hidalgo CA Barbette J Santhanam B, 2012 Proto-genes and *de novo* gene birth. Nature 487:370–374. 22722833 10.1038/nature11184 PMC3401362

[msw083-B8] Casal G Clemente SC Matos P Knoff M Matos E Abdel-Baki AA Azevedo C. 2012 Redefining the genus *Spraguea* based on ultrastructural and phylogenetic data from *Spraguea gastrophysus* n. sp. (phylum Microsporidia), a parasite found in *Lophius gastrophysus* (Teleostei) from Brazil. Parasitol Res. 111:79–88. 22223036 10.1007/s00436-011-2803-8

[msw083-B9] Cornman RS Chen YP Schatz MC Street C Zhao Y Desany B Egholm M Hutchison S Pettis JS Lipkin WI, 2009 Genomic analyses of the microsporidian *Nosema ceranae*, an emergent pathogen of honey bees. PLoS Pathog. 5:e1000466. 19503607 10.1371/journal.ppat.1000466 PMC2685015

[msw083-B10] Corradi N Pombert JF Farinelli L Didier ES Keeling PJ. 2010 The complete sequence of the smallest known nuclear genome from the microsporidian *Encephalitozoon intestinalis.* Nat Commun. 1:77. 20865802 10.1038/ncomms1082 PMC4355639

[msw083-B11] Criscuolo A Gribaldo S. 2010 BMGE (Block Mapping and Gathering with Entropy): a new software for selection of phylogenetic informative regions from multiple sequence alignments. BMC Evol Biol. 10:210. 20626897 10.1186/1471-2148-10-210 PMC3017758

[msw083-B12] Crusoe MR Alameldin HF Awad S Boucher E Caldwell A Cartwright R Charbonneau A Constantinides B Edvenson G Fay S, 2015 The khmer software package: enabling efficient nucleotide sequence analysis. F1000Res 4:900. 26535114 10.12688/f1000research.6924.1 PMC4608353

[msw083-B13] Cuomo CA Desjardins CA Bakowski MA Goldberg J Ma AT Becnel JJ Didier ES Fan L Heiman DI Levin JZ, 2012 Microsporidian genome analysis reveals evolutionary strategies for obligate intracellular growth. Genome Res. 22:2478–2488. 22813931 10.1101/gr.142802.112 PMC3514677

[msw083-B14] Dehal P Boore JL. 2005 Two rounds of whole genome duplication in the ancestral vertebrate. PLoS Biol. 3:e314. 16128622 10.1371/journal.pbio.0030314 PMC1197285

[msw083-B15] Desjardins CA Sanscrainte ND Goldberg JM Heiman D Young S Zeng Q Madhani HD Becnel JJ Cuomo CA. 2015 Contrasting host-pathogen interactions and genome evolution in two generalist and specialist microsporidian pathogens of mosquitoes. Nat Commun. 6:7121. 25968466 10.1038/ncomms8121 PMC4435813

[msw083-B16] Didier ES Didier PJ Snowden KF Shadduck JA. 2000 Microsporidiosis in mammals. Microbes Infect. 2:709–720. 10884622 10.1016/s1286-4579(00)00354-3

[msw083-B17] Didier ES Weiss LM. 2011 Microsporidiosis: not just in AIDS patients. Curr Opin Infect Dis. 24:490–495. 21844802 10.1097/QCO.0b013e32834aa152 PMC3416021

[msw083-B18] Edgar RC. 2004 MUSCLE: multiple sequence alignment with high accuracy and high throughput. Nucleic Acids Res. 32:1792–1797. 15034147 10.1093/nar/gkh340 PMC390337

[msw083-B19] Edger PP Pires JC. 2009 Gene and genome duplications: the impact of dosage-sensitivity on the fate of nuclear genes. Chromosome Res. 17:699–717. 19802709 10.1007/s10577-009-9055-9

[msw083-B20] Force A Lynch M Pickett FB Amores A Yan YL Postlethwait J. 1999 Preservation of duplicate genes by complementary, degenerative mutations. Genetics 151:1531–1545. 10101175 10.1093/genetics/151.4.1531 PMC1460548

[msw083-B21] Freeman MA Yokoyama H Ogawa K. 2004 A microsporidian parasite of the genus *Spraguea* in the nervous tissues of the Japanese anglerfish *Lophius litulon.* Folia Parasitol. 51:167–176. 15357394 10.14411/fp.2004.020

[msw083-B22] Freeman MA Yokoyama H Osada A Yoshida T Yamanobe A Ogawa K. 2011 *Spraguea* (Microsporida: Spraguidae) infections in the nervous system of the Japanese anglerfish, *Lophius litulon* (Jordan), with comments on transmission routes and host pathology. J Fish Dis. 34:445–452. 21545438 10.1111/j.1365-2761.2011.01255.x

[msw083-B23] Haag KL Traunecker E Ebert D. 2013 Single-nucleotide polymorphisms of two closely related microsporidian parasites suggest a clonal population expansion after the last glaciation. Mol Ecol. 22:314–326. 23163569 10.1111/mec.12126

[msw083-B24] Heinz E Williams TA Nakjang S Noel CJ Swan DC Goldberg AV Harris SR Weinmaier T Markert S Becher D, 2012 The genome of the obligate intracellular parasite *Trachipleistophora hominis*: new insights into microsporidian genome dynamics and reductive evolution. PLoS Pathog. 8:e1002979. 23133373 10.1371/journal.ppat.1002979 PMC3486916

[msw083-B25] Hershberg R Petrov DA. 2010 Evidence that mutation is universally biased towards AT in bacteria. PLoS Genet. 6:e1001115. 20838599 10.1371/journal.pgen.1001115 PMC2936535

[msw083-B26] Hildebrand F Meyer A Eyre-Walker A. 2010 Evidence of selection upon genomic GC-content in bacteria. PLoS Genet. 6:e1001107. 20838593 10.1371/journal.pgen.1001107 PMC2936529

[msw083-B27] Hislop JRG Gallego A Heath MR Kennedy FM Reeves SA Wright PJ. 2001 A synthesis of the early life history of the anglerfish, *Lophius piscatorius* (Linnaeus, 1758) in northern British waters. ICES J Mar Sci. 58:70–86.

[msw083-B28] Hollister WS Canning EU Weidner E Field AS Kench J Marriott DJ. 1996 Development and ultrastructure of *Trachipleistophora hominis* n.g., n.sp. after in vitro isolation from an AIDS patient and inoculation into athymic mice. Parasitology 112(Pt 1):143–154. 8587798 10.1017/s0031182000065185

[msw083-B29] Hunter JD. 2007 Matplotlib: A 2D graphics environment. Comput Sci Eng. 9:90–95.

[msw083-B30] Hyatt D Chen GL Locascio PF Land ML Larimer FW Hauser LJ. 2010 Prodigal: prokaryotic gene recognition and translation initiation site identification. BMC Bioinformatics 11:119. 20211023 10.1186/1471-2105-11-119 PMC2848648

[msw083-B31] Katinka MD Duprat S Cornillot E Metenier G Thomarat F Prensier G Barbe V Peyretaillade E Brottier P Wincker P, 2001 Genome sequence and gene compaction of the eukaryote parasite *Encephalitozoon cuniculi.* Nature 414:450–453. 11719806 10.1038/35106579

[msw083-B32] Kelley DR Schatz MC Salzberg SL. 2010 Quake: quality-aware detection and correction of sequencing errors. Genome Biol. 11:R116. 21114842 10.1186/gb-2010-11-11-r116 PMC3156955

[msw083-B33] Kent ML Elliott DG Groff JM Hedrick RP. 1989 *Loma salmonae* (Protozoa, Microspora) Infections in Seawater Reared Coho Salmon *Oncorhynchus kisutch.* Aquaculture 80:211–222.

[msw083-B34] Kidwell MG. 2002 Transposable elements and the evolution of genome size in eukaryotes. Genetica 115:49–63. 12188048 10.1023/a:1016072014259

[msw083-B35] Kimura M. 1980 A simple method for estimating evolutionary rates of base substitutions through comparative studies of nucleotide sequences. J Mol Evol. 16:111–120. 7463489 10.1007/BF01731581

[msw083-B36] Kryazhimskiy S Plotkin JB. 2008 The population genetics of dN/dS. PLoS Genet. 4:e1000304. 19081788 10.1371/journal.pgen.1000304 PMC2596312

[msw083-B37] Lartillot N Philippe H. 2004 A Bayesian mixture model for across-site heterogeneities in the amino-acid replacement process. Mol Biol Evol. 21:1095–1109. 15014145 10.1093/molbev/msh112

[msw083-B38] Lartillot N Rodrigue N Stubbs D Richer J. 2013 PhyloBayes MPI: phylogenetic reconstruction with infinite mixtures of profiles in a parallel environment. Syst Biol. 62:611–615. 23564032 10.1093/sysbio/syt022

[msw083-B39] Leigh JW Susko E Baumgartner M Roger AJ. 2008 Testing congruence in phylogenomic analysis. Syst Biol. 57:104–115. 18288620 10.1080/10635150801910436

[msw083-B40] Liu S Liu Y Yang X Tong C Edwards D Parkin IA Zhao M Ma J Yu J Huang S, 2014 The *Brassica oleracea* genome reveals the asymmetrical evolution of polyploid genomes. Nat Commun. 5:3930. 24852848 10.1038/ncomms4930 PMC4279128

[msw083-B41] Lunter G Goodson M. 2011 Stampy: a statistical algorithm for sensitive and fast mapping of Illumina sequence reads. Genome Res. 21:936–939. 20980556 10.1101/gr.111120.110 PMC3106326

[msw083-B42] Lynch M. 2010 Rate, molecular spectrum, and consequences of human mutation. Proc Natl Acad Sci U S A. 107:961–968. 20080596 10.1073/pnas.0912629107 PMC2824313

[msw083-B43] Lynch M Sung W Morris K Coffey N Landry CR Dopman EB Dickinson WJ Okamoto K Kulkarni S Hartl DL, 2008 A genome-wide view of the spectrum of spontaneous mutations in yeast. Proc Natl Acad Sci U S A. 105:9272–9277. 18583475 10.1073/pnas.0803466105 PMC2453693

[msw083-B44] Magoc T Salzberg SL. 2011 FLASH: fast length adjustment of short reads to improve genome assemblies. Bioinformatics 27:2957–2963. 21903629 10.1093/bioinformatics/btr507 PMC3198573

[msw083-B45] Mansour L Ben Hassine OK Vivares CP Cornillot E. 2013 *Spraguea lophii* (Microsporidia) parasite of the teleost fish, *Lophius piscatorius* from Tunisian coasts: evidence for an extensive chromosome length polymorphism. Parasitol Int. 62:66–74. 23059913 10.1016/j.parint.2012.09.007

[msw083-B46] McLysaght A Hokamp K Wolfe KH. 2002 Extensive genomic duplication during early chordate evolution. Nat Genet. 31:200–204. 12032567 10.1038/ng884

[msw083-B47] Nakjang S Williams TA Heinz E Watson AK Foster PG Sendra KM Heaps SE Hirt RP Martin Embley T. 2013 Reduction and expansion in microsporidian genome evolution: new insights from comparative genomics. Genome Biol Evol. 5:2285–2303. 24259309 10.1093/gbe/evt184 PMC3879972

[msw083-B48] Oliphant TE. 2007 Python for scientific computing. Comput Sci Eng. 9:10–20.

[msw083-B49] Pan G Xu J Li T Xia Q Liu SL Zhang G Li S Li C Liu H Yang L, 2013 Comparative genomics of parasitic silkworm microsporidia reveal an association between genome expansion and host adaptation. BMC Genomics 14:186. 23496955 10.1186/1471-2164-14-186 PMC3614468

[msw083-B50] Papp B Pal C Hurst LD. 2003 Dosage sensitivity and the evolution of gene families in yeast. Nature 424:194–197. 12853957 10.1038/nature01771

[msw083-B51] Paradis E Claude J Strimmer K. 2004 APE: analyses of phylogenetics and evolution in R language. Bioinformatics 20:289–290. 14734327 10.1093/bioinformatics/btg412

[msw083-B52] Pelin A Selman M Aris-Brosou S Farinelli L Corradi N. 2015 Genome analyses suggest the presence of polyploidy and recent human-driven expansions in eight global populations of the honeybee pathogen *Nosema ceranae.* Environ Microbiol. 17:4443–4458. 25914091 10.1111/1462-2920.12883

[msw083-B53] Peuvel I Delbac F Metenier G Peyret P Vivares CP. 2000 Polymorphism of the gene encoding a major polar tube protein PTP1 in two microsporidia of the genus *Encephalitozoon.* Parasitology 121(Pt 6):581–587. 11155928 10.1017/s0031182000006910

[msw083-B54] Peyretaillade E Goncalves O Terrat S Dugat-Bony E Wincker P Cornman RS Evans JD Delbac F Peyret P. 2009 Identification of transcriptional signals in *Encephalitozoon cuniculi* widespread among Microsporidia phylum: support for accurate structural genome annotation. BMC Genomics 10:607. 20003517 10.1186/1471-2164-10-607 PMC2803860

[msw083-B55] Pombert JF Xu J Smith DR Heiman D Young S Cuomo CA Weiss LM Keeling PJ. 2013 Complete genome sequences from three genetically distinct strains reveal high intraspecies genetic diversity in the microsporidian *Encephalitozoon cuniculi.* Eukaryot Cell 12:503–511. 23291622 10.1128/EC.00312-12 PMC3623445

[msw083-B56] Raymond M Rousset F. 1995 Genepop (Version-1.2)—population-genetics software for exact tests and ecumenicism. J Hered. 86:248–249.

[msw083-B57] Rousset F. 2008 GENEPOP ' 007: a complete re-implementation of the GENEPOP software for Windows and Linux. Mol Ecol Resources 8:103–106. 10.1111/j.1471-8286.2007.01931.x 21585727

[msw083-B58] Saito R Smoot ME Ono K Ruscheinski J Wang PL Lotia S Pico AR Bader GD Ideker T. 2012 A travel guide to Cytoscape plugins. Nat Methods 9:1069–1076. 23132118 10.1038/nmeth.2212 PMC3649846

[msw083-B59] Scannell DR Byrne KP Gordon JL Wong S Wolfe KH. 2006 Multiple rounds of speciation associated with reciprocal gene loss in polyploid yeasts. Nature 440:341–345. 16541074 10.1038/nature04562

[msw083-B60] Scannell DR Frank AC Conant GC Byrne KP Woolfit M Wolfe KH. 2007 Independent sorting-out of thousands of duplicated gene pairs in two yeast species descended from a whole-genome duplication. Proc Natl Acad Sci U S A. 104:8397–8402. 17494770 10.1073/pnas.0608218104 PMC1895961

[msw083-B61] Stentiford GD Feist SW Stone DM Bateman KS Dunn AM. 2013 Microsporidia: diverse, dynamic, and emergent pathogens in aquatic systems. Trends Parasitol. 29:567–578. 24091244 10.1016/j.pt.2013.08.005

[msw083-B62] Takvorian PM Cali A. 1986 The ultrastructure of spores (Protozoa: Microsporida) from *Lophius americanus*, the angler fish. J Protozool. 33:570–575. 3795144 10.1111/j.1550-7408.1986.tb05664.x

[msw083-B63] Tatusov RL Fedorova ND Jackson JD Jacobs AR Kiryutin B Koonin EV Krylov DM Mazumder R Mekhedov SL Nikolskaya AN, 2003 The COG database: an updated version includes eukaryotes. BMC Bioinformatics 4:41. 12969510 10.1186/1471-2105-4-41 PMC222959

[msw083-B64] Tsaousis AD Kunji ER Goldberg AV Lucocq JM Hirt RP Embley TM. 2008 A novel route for ATP acquisition by the remnant mitochondria of *Encephalitozoon cuniculi.* Nature 453:553–556. 18449191 10.1038/nature06903

[msw083-B65] Vavra J Lukes J. 2013 Microsporidia and ‘the art of living together’. Adv Parasitol. 82:253–319. 23548087 10.1016/B978-0-12-407706-5.00004-6

[msw083-B66] Walsh JB. 1995 How often do duplicated genes evolve new functions? Genetics 139:421–428. 7705642 10.1093/genetics/139.1.421 PMC1206338

[msw083-B67] Watson AK Williams TA Williams BA Moore KA Hirt RP Embley TM. 2015 Transcriptomic profiling of host-parasite interactions in the microsporidian *Trachipleistophora hominis.* BMC Genomics 16:983. 26589282 10.1186/s12864-015-1989-z PMC4654818

[msw083-B68] Williams BA Hirt RP Lucocq JM Embley TM. 2002 A mitochondrial remnant in the microsporidian *Trachipleistophora hominis.* Nature 418:865–869. 12192407 10.1038/nature00949

[msw083-B69] Williams BA Slamovits CH Patron NJ Fast NM Keeling PJ. 2005 A high frequency of overlapping gene expression in compacted eukaryotic genomes. Proc Natl Acad Sci U S A. 102:10936–10941. 16037215 10.1073/pnas.0501321102 PMC1182411

[msw083-B70] Xiao L Li L Moura H Sulaiman IM Lal AA Gatti S Scaglia M Didier ES Visvesvara GS. 2001 Genotyping *Encephalitozoon* parasites using multilocus analyses of genes with repetitive sequences. J Eukaryot Microbiol. Suppl:63s–65s. 11906081 10.1111/j.1550-7408.2001.tb00454.x

[msw083-B71] Yokoyama H Miyazaki Y Yoshinaga T. 2013 Microsporidian encephalomyelitis in cultured yellowtail *Seriola quinqueradiata.* Fish Pathol. 48:119–125.

