# Supplementary material for: A Recent Whole-Genome Duplication Divides Populations of a Globally Distributed Microsporidian
Source: Mol Biol Evol. 2016 Apr 27;33(8):2002–15. doi: 10.1093/molbev/msw083 (PMC4948709; doi:10.1093/molbev/msw083)
Supplement: Supplementary Data [file supp_33_8_2002__index.html]

A Recent Whole-Genome Duplication Divides Populations of a Globally Distributed Microsporidian — A Recent Whole-Genome Duplication Divides Populations of a Globally Distributed Microsporidian — Supplementary Data 

# A Recent Whole-Genome Duplication Divides Populations of a Globally Distributed Microsporidian

## Supplementary Data

files

- Supplementary Data - xlsx file
- Supplementary Data - pdf file
