# Supplementary material for: A Recent Whole-Genome Duplication Divides Populations of a Globally Distributed Microsporidian
Source: Mol Biol Evol. 2016 Apr 27;33(8):2002–15. doi: 10.1093/molbev/msw083 (PMC4948709; doi:10.1093/molbev/msw083)
Supplement: Supplementary Data [file supp_msw083_Supplementaryfiles.pdf]

Supplementary files: A recent whole-genome duplication divides populations of a globally-distributed microsporidian parasite.

Tom A. Williams[1], Sirintra Nakjang[1], Scott E. Campbell[2], Mark A. Freeman[3], Matthías Eydal[4], Karen Moore[2], Robert P. Hirt[1], T. Martin Embley[1], Bryony A. P. Williams[2*]

Supplementary Table 1: Gene content/genome completeness analyses for 11 de novo assemblies.

**Population**	**Isolate**	**Assembly Size (bp)**	**Number of predicted protein-coding genes (CDS)**	**Number of nucleotides (bp) in predicted CDS**	**Predicted CDS with blastp hits to proteins from reference *S. lophii* genome***	**Number of nucleotides (bp) in predicted CDSs homologous to reference *S. lophii***	**Number of unique hits to *S. lophii* reference proteome (2499 predicted CDS)***
Celtic Sea	Celtic Deep	5,774,772	3748	3166202	2642 (70.5%)	2865544	2275
Celtic Sea	EM120	6,118,728	4333	3348068	2698 (62.3%)	2784743	2161
Celtic Sea	RA12034	5,811,366	3827	3184069	2648 (69.2%)	2871013	2274
Celtic Sea	North Atlantic	5,860,603	3854	3204607	2661 (69%)	2881014	2275
North America	New Brunswick (NB) 1	7,734,566	7427	4186003	5170 (69.6%)	3609665	2295
North America	NB4	7,741,808	7406	4172155	5108 (69%)	3596206	2307
North America	NB8	7,758,278	7445	4205221	5203 (70%)	3642824	2310
North America	NB9	7,712,656	7428	4172055	5175 (69.7%)	3619176	2316
North America	New Jersey (NJ) JS61	7,753,355	7720	4199891	3961 (51.3%)	2723192	1969
North America	NJ RM18	7,719,948	7688	4183132	3978 (51.7%)	2708934	1882
North America	NJ RW92	7,706,796	7589	4177696	3969 (52.3%)	2727879	1908

blastp cut-off evalue ≤ 0.0001

Supplementary Table 3: Comparison of AT content in microsporidian coding and intergenic regions. Fisher's exact tests were performed based on the counts of AT and GC nucleotides in coding and intergenic regions for each species. Definitions of coding and intergenic sequence were based on the assemblies downloaded from MicrosporidiaDB, or on our own Prodigal-based annotations. Note that while the difference between the AT content of coding and intergenic regions is significant in all cases, in two of the species (*E. bieneusi* and *N. bombycis*) the direction of the trend is opposite to that in the other 12 cases: in these species, coding AT exceeds that of intergenic AT.

Species	AT% (coding)	AT% (whole genome)	AT% (intergenic)	P-value (Coding vs. intergenic AT, Fisher's exact test)
Spraguea lophii "Celtic Deep"	74.7	76.7	79.2	0
Encephalitozoon cuniculi	52.4	52.6	54	0
Encephalitozoon hellem	56.6	56.1	60	0
Encephalitozoon romaleae	59.1	59.6	63.4	0
Nosema ceranae	72.6	74.7	75.6	0
Nosema bombycis	69.4	69.1	69.1	9.12 x 10-25
Enterocytozoon bieneusi	67.9	66.2	62.8	0
Vittaforma corneae	61.8	63.5	67.1	0
Trachipleistophora hominis	59.4	65.9	69.5	0
Vavraia culicis	57.3	60.2	62.9	0
Nematocida sp. ERTm2	59	61.7	67.4	0
Nematocida parisii ERTm1	63	65.5	72.3	0
Nematocida parisii ERTm3	62.9	65.5	71.4	0

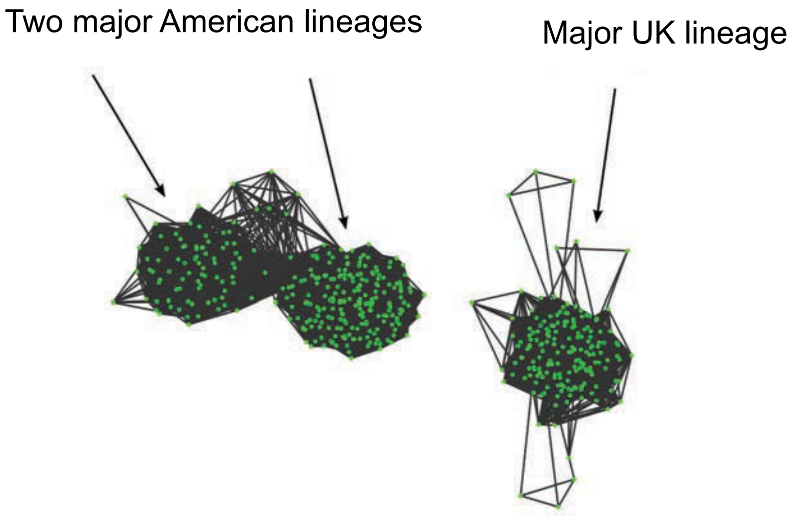

Supplementary Figure 1: Non-LTR retrotransposon similarity network. Similarity clustering of fragmented transposon sequences reveals three major lineages of non-LTR retrotransposons in *Spraguea*, all of which were represented in the common ancestor of the North American and Celtic Sea populations. The presence of sequences from each of the three clusters in both the North American and Celtic Sea populations suggests that all three clusters were already present in the common ancestor of the two populations. Although transposon content has fluctuated in both lineages since the divergence of the two populations, the differences largely reflect differential reduction and expansion of transposon subfamilies, and they do not make a major contribution to the conserved difference in genome size between the North American and Celtic Sea populations.

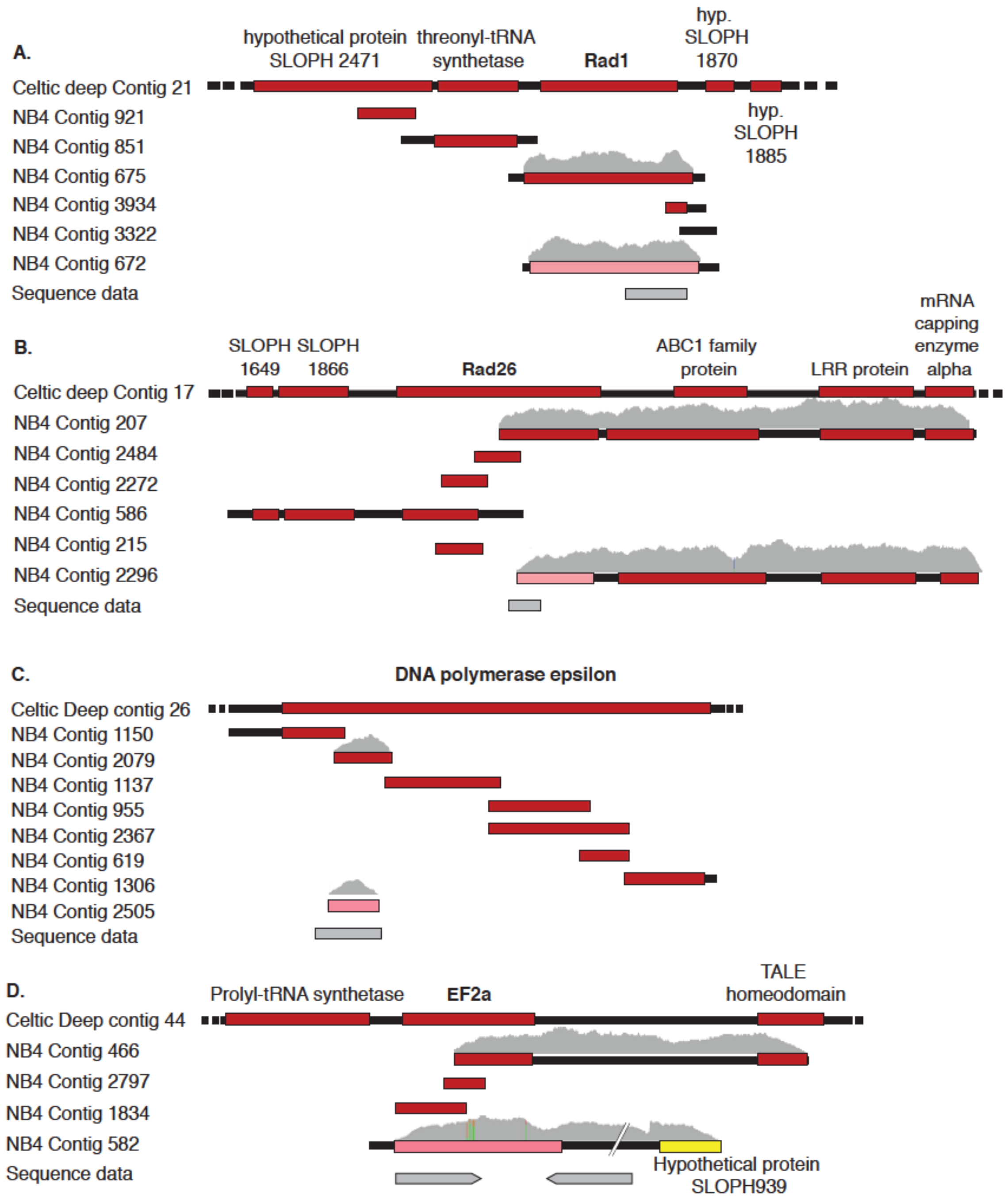

Supplementary Figure 2: Examples of fragmented genes in the genome of North American samples with their genomic context. Red boxes indicate intact genes, pink boxes indicate pseudogenes. (A) DNA repair protein RAD1 is a 2109 bp open reading frame. In Celtic Deep it is a part of a 95382 bp contig. In NB4 several short contigs match this area of the genome. One of these covers the complete DNA repair protein RAD1 ORF. Another contig represents part of a non-functional copy of the same gene. A fragment of this frameshifted gene was amplified by PCR and sequenced. Grey charts show coverage (max. 53 for contig 675, max 64 for contig 672). (B) RAD26 is a 3027 bp open reading frame. In Celtic Deep it is a part of a 102889 bp contig. For RAD26 in NB4 there are several contigs representing this area. One set of these contigs can be assembled to create a contig for 6 contiguous open reading frames. Two other contigs are similar to this area but not identical. One of these contigs (215) matches four of the genes, but a frame shift is created in the RAD26 gene. We amplified part of the contig by PCR and sequenced it. Grey charts show coverage (max. 79 for contig 207, max 81 for contig 215). (C) DNA polymerase epsilon is a 5261 bp open reading frame. In Celtic deep it is a part of a 80999 bp contig. In NB4 several short contigs match this area of the genome. Some of these can be assembled to make a complete ORF. Another contig represents part of a non-functional copy of the same gene (creating multiple stop codons). This frameshifted genes was amplified by PCR and sequenced. Grey charts show coverage (max. 48 for contig 2079, max 35 for contig 2505). (D) EF2a is a 1332 bp open reading frame. In Celtic deep it is a part of a 38300 bp contig. In NB4 several short contigs match this area of the genome. Some of these can be assembled to cover a complete ORF for EF2a. Another contig bears a non-functional copy of the same gene. In addition, this fragment contain a stretch of DNA not found in the other EF2a contigs from Celtic Deep or NB4. This is suggestive of segmental, rather than whole chromosome duplication - an increase in rearrangements may be facilitated by the redundancy resulting from a whole genome duplication. Part of this stretch of DNA was amplified by PCR and was shown to only be present in the NB4 and not Celtic Deep sample (PCR result shown). Grey charts show coverage (max. 66 for contig 466, max 62 for contig 672).

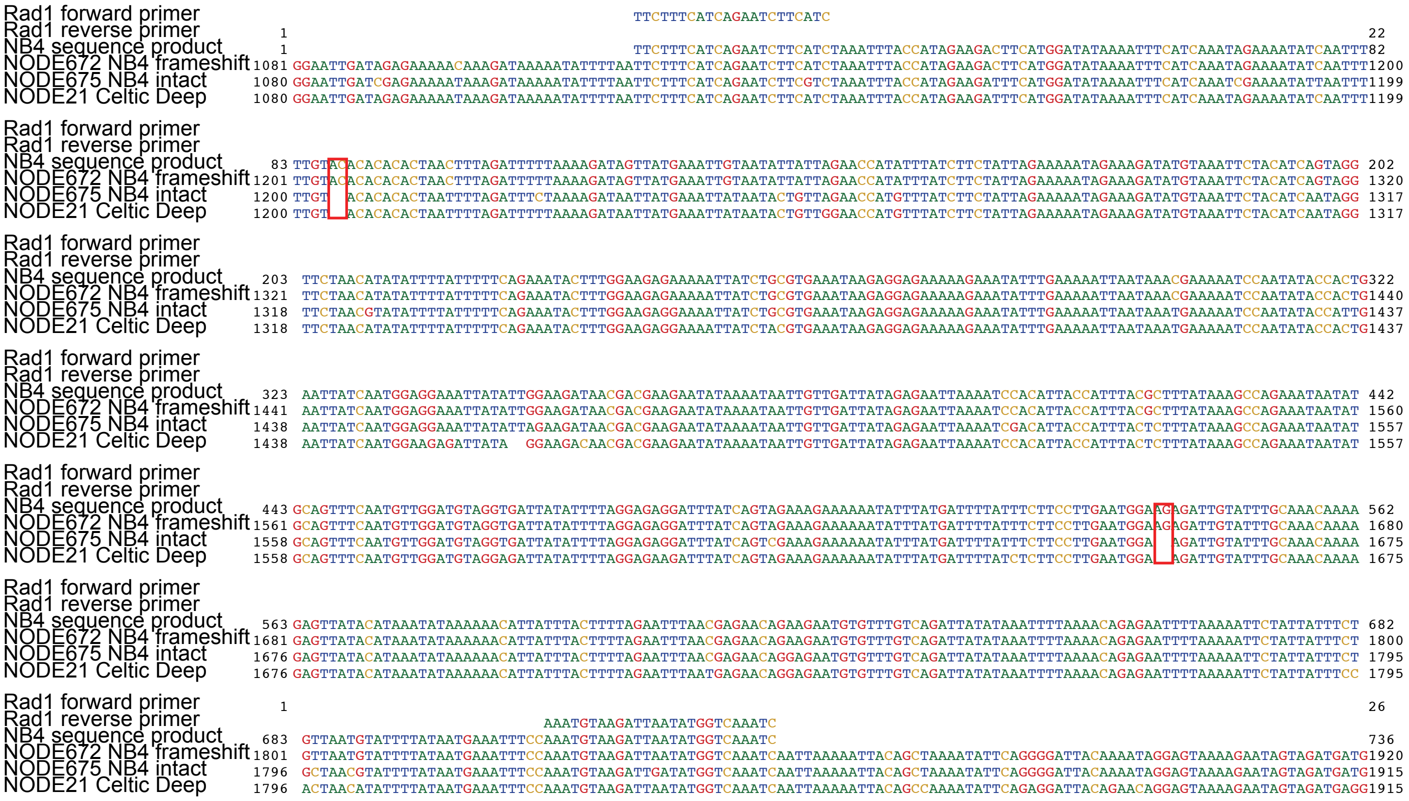

Supplementary Figure 3: Alignment of RAD1 PCR primers and sequencing product to all RAD1 gene copies from Celtic Deep and NB4 populations. Sites of insertions causing frame shifts are indicated by red boxes.

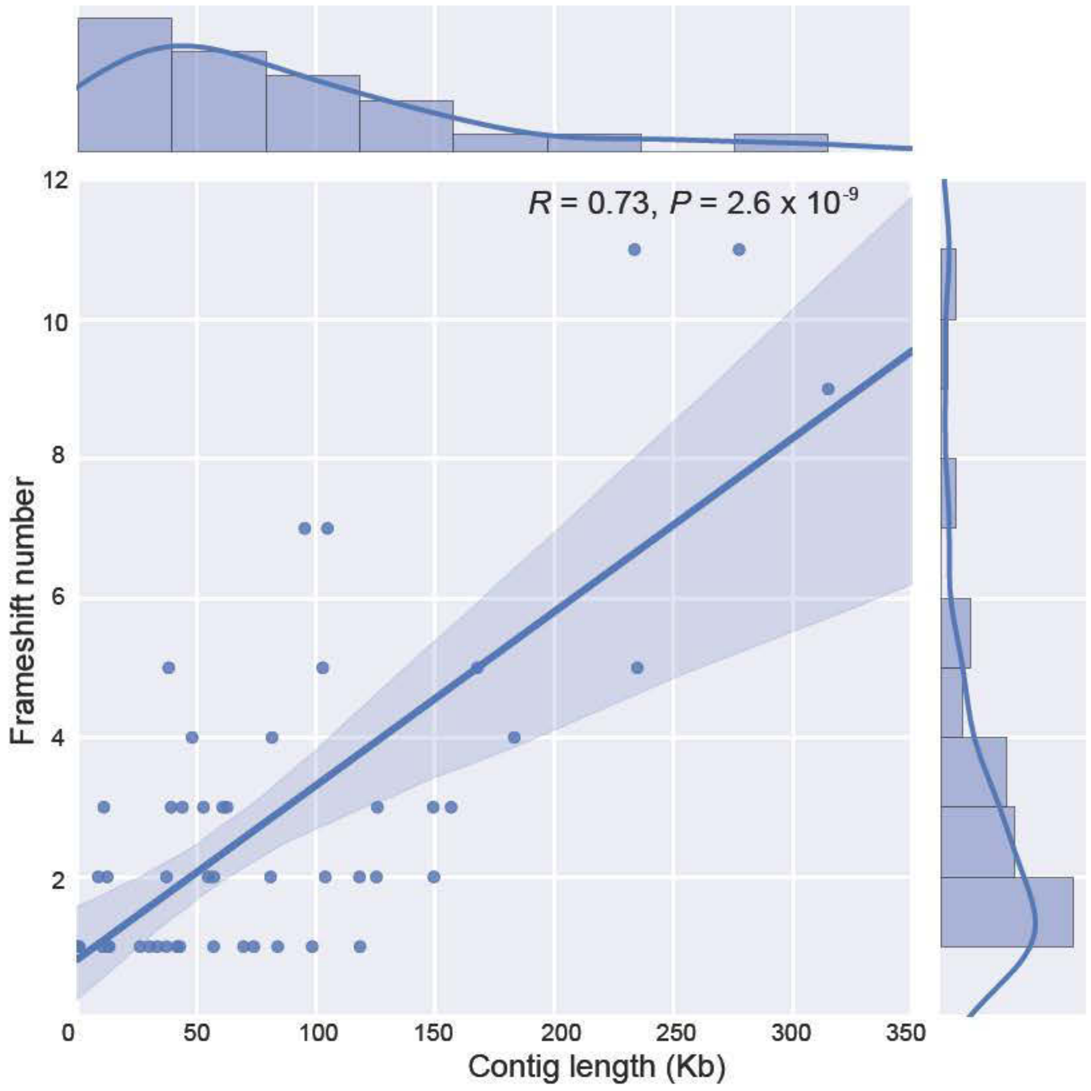

Supplementary Figure 4: Locations of Celtic Deep orthologues of genes frameshifted in the North American population. The distribution is apparently random, with the number of orthologues on each Celtic Deep contig roughly proportional to contig length.

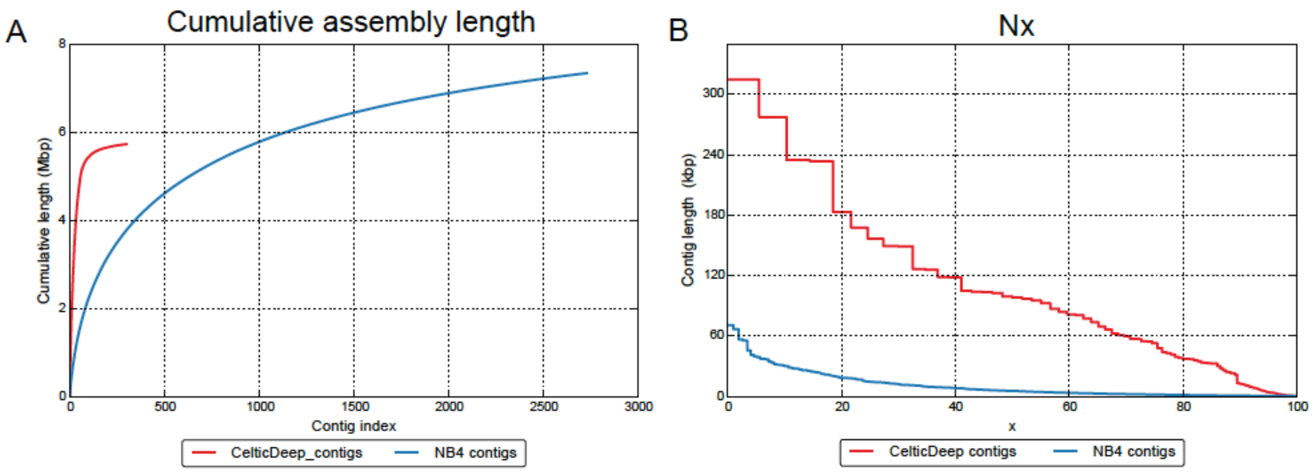

Supplementary Figure 5: North American *Spraguea* assemblies contain a large number of additional short contigs. All the North American assemblies, regardless of sequencing run, contain an additional ~2 Mb of sequence spread across a large number of small contigs. This is reflected in both (A) the cumulative assembly lengths and (B) the Nx plots for the assemblies, and may arise from the heterozygosity found in North American but not Celtic Sea isolates. Here, the results from two representative assemblies are shown: "Celtic Deep" for the Celtic Sea isolates, and "New Brunswick 4" for the North American isolates.

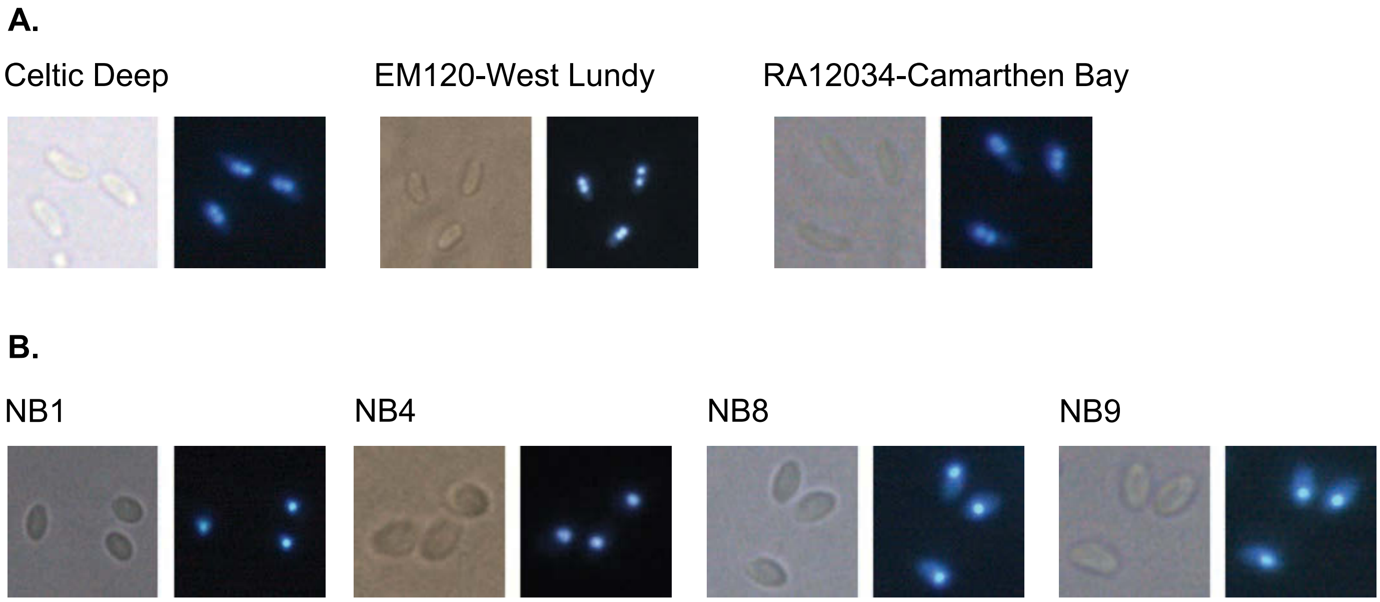

Supplementary Figure 6: Uni- and dinucleate *Spraguea* spores. Images of spores showing the nuclear configuration of samples collected at (A) Site 1 Celtic Sea (UK) and (B) North American (Canada). In each case a brightfield image is shown to the left and DAPI-stained cells under UV illumination are shown to the right; unlike in a number of other microsporidians, DAPI can be used to detect nuclei in *Spraguea* spores.

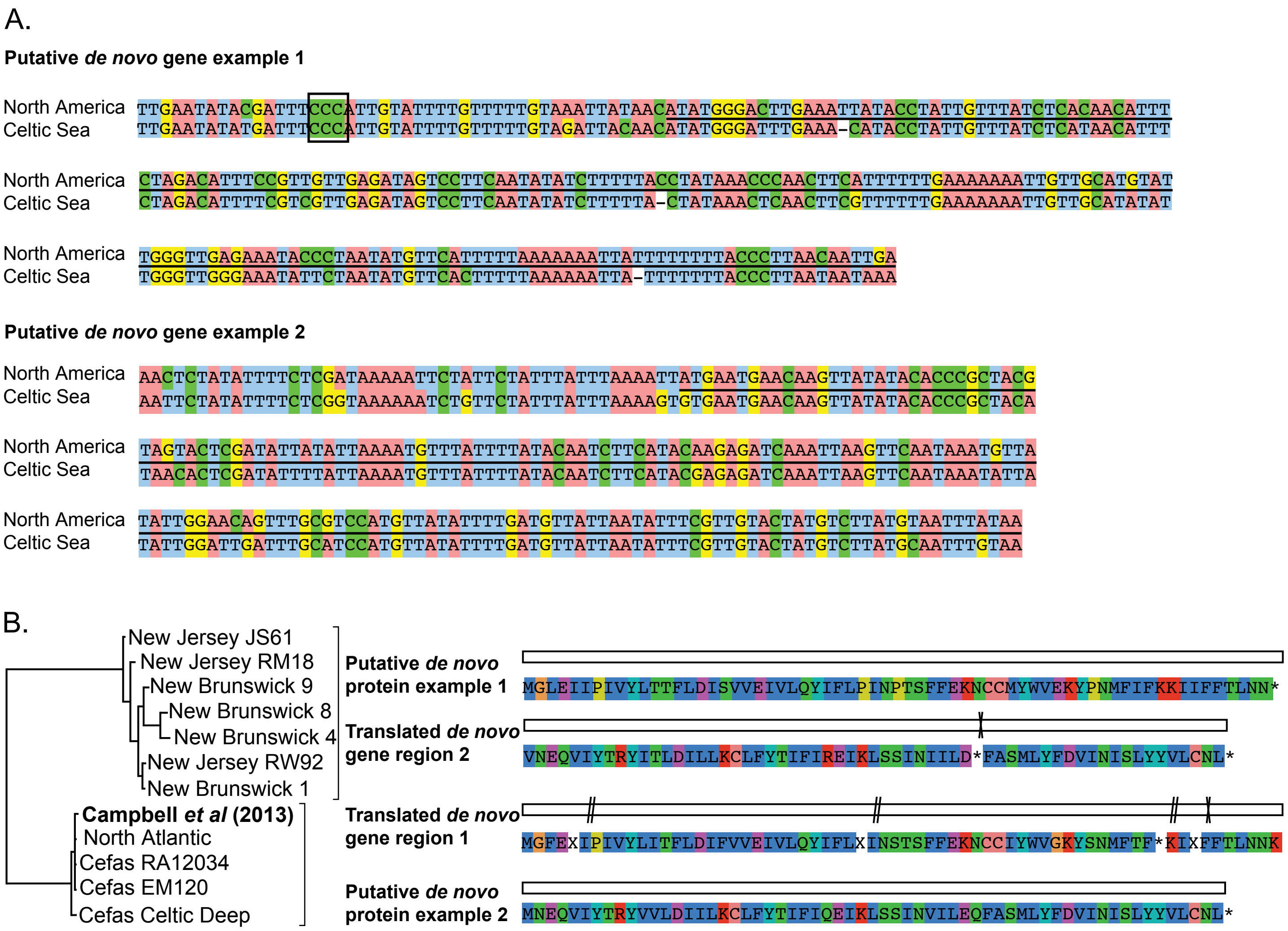

Supplementary Figure 7: Illustrations of two genome areas encoding potential *de novo* genes. (A) DNA alignment of relevant areas from North East and North American (In this case this area shows 100% identity among populations). Boxed is a CCC motif that could act as a promoter for transcription. (B) Translation of the respective areas showing intact short ORFs in one population but not the other. Oblique lines indicate sites of deletion and crosses indicate sites of stop codons.
